# Nanomaterials at the forefront of antimicrobial therapy by photodynamic and photothermal strategies

**DOI:** 10.1016/j.mtbio.2024.101354

**Published:** 2024-11-22

**Authors:** Ling Mei, Yifan Zhang, Kaixi Wang, Sijing Chen, Tao Song

**Affiliations:** a Engineering Research Center for Pharmaceuticals and Equipments of Sichuan Province, Sichuan Industrial Institute of Antibiotics, School of Pharmacy, Chengdu University, Chengdu, 610106, China; b Sichuan Electric Power Hospital, Chengdu, Sichuan Province, China

**Keywords:** Nanomaterials, Photothermal therapy, Photodynamic therapy, Antimicrobial therapy

## Abstract

In the face of the increasing resistance of microorganisms to traditional antibiotics, the development of innovative treatment methods is becoming increasingly urgent. Nanophototherapy technology can precisely target the infected area and achieve synergistic antibacterial effects in multiple modes. This phototherapy method has shown significant efficacy in treating diseases caused by drug-resistant bacteria, especially in the elimination of biofilms, where it has demonstrated strong dissolution capabilities. PTT utilizes photothermal agents to convert near-infrared light into heat, effectively killing bacteria and promoting tissue regeneration. Similarly, PDT utilizes photosensitizers, which produce reactive oxygen species (ROS) when activated by light, destroying the structure and function of bacterial cells. This review summarizes photothermal agents and photosensitizers used for antibacterial purposes. In conducting our literature review, we employed a systematic approach to ensure a comprehensive and representative selection of studies. Additionally, this article explores the potential of phototherapy in regulating wound microenvironments, promoting wound healing, and activating the immune system. Nanophototherapeutic materials show great potential for application in antibacterial treatment and are expected to provide innovative solutions for drug-resistant bacterial infections that traditional antibiotics are struggling to address.

## Graphical abstract

**Figure:**
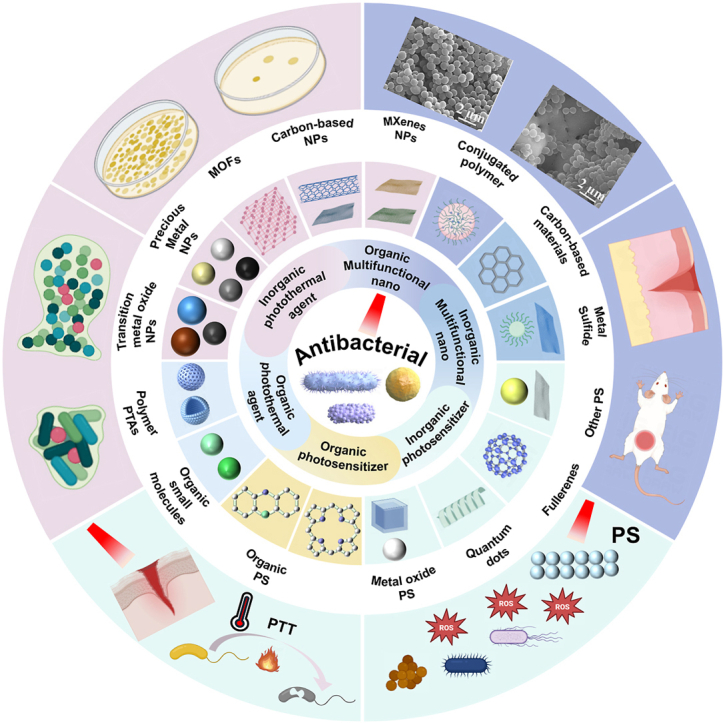

## 1 Introduction

### 1.1 Global threat of bacterial infections

Antimicrobial resistance (AMR) has been recognized by the World Health Organization (WHO) as a looming global health crisis, with projections indicating that it could cause up to 10 million deaths per year by 2050. AMR is primarily driven by the overuse and misuse of antibiotics in healthcare, agriculture, and livestock farming. These actions create selective pressure on bacteria, allowing resistant strains to survive and multiply. This selective pressure accelerates the evolution of resistance and increases the likelihood of genetic mutations and horizontal gene transfer, further spreading resistance genes within bacterial populations. As a result, infections caused by resistant bacteria often lead to treatment failures, with standard antibiotics becoming ineffective. This forces the use of more expensive, potent, and sometimes more toxic alternatives, which not only increases the cost of treatment but also prolongs hospital stays and recovery times. In severe cases, patients may face complications or even death due to the lack of effective therapeutic options. Moreover, the increased need for intensive care and additional medical interventions places a heavy burden on healthcare systems.

Currently, nearly all classes of antibiotics, despite their differing mechanisms of action, are facing the challenge of antimicrobial resistance (AMR). For example, beta-lactams, which target bacterial cell wall synthesis, have encountered widespread resistance due to the production of beta-lactamases. Macrolides and aminoglycosides, both of which inhibit protein synthesis, are also compromised by resistance mechanisms such as target site modifications and active efflux. Even quinolones, which disrupt bacterial DNA replication, have been met with resistance through mutations in the target enzymes and increased drug efflux. This widespread emergence of resistance, regardless of the antibiotic's mechanism of action, emphasizes the need for innovative antimicrobial strategies to overcome or bypass these resistance mechanisms.

Infections occur when pathogenic or opportunistic bacteria infiltrate the bloodstream, proliferate, and generate toxins and other metabolic byproducts, culminating in severe systemic infections. These bacterial diseases represent a substantial risk to worldwide health, manifesting in various serious conditions such as lung infections, tuberculosis, cholera, blood poisoning, meningitis, bone infections, and soft tissue infections. The colonization of bacteria on medical devices and implants constitutes an increasing challenge, frequently resulting in device failure and an elevated risk of subsequent complications. The management of bacterial infections generally involves the use of antibacterial agents. Antimicrobials, predominantly antibiotics, are derived from specific chemically synthesized substances obtained through the cultivation of microorganisms, including bacteria, actinomycetes, and fungi. This category encompasses a diverse array of compounds such as antibiotics, sulfonamides, imidazoles, nitroimidazoles, and quinolones. Antibiotics function through unique mechanisms to eliminate or suppress bacterial growth. The bactericidal effects of these drugs are mainly achieved through five pathways: preventing the assembly of bacterial cell walls, engaging with the cell membrane, interrupting process of protein formation, and inhibiting the processes of genetic replication and transcription. The advent and utilization of antibiotics have marked a pivotal advancement in medical history. Nevertheless, the extensive clinical deployment of antibiotics precipitated the swift rise in drug resistance [1]. Microorganisms face selective pressures that drive resistance and the acquisition of adaptive mutations or genes, particularly when antimicrobials are inappropriately or excessively used across healthcare, veterinary, and agricultural contexts [2]. As bacteria evolve or acquire resistance, managing infections such as those caused by gram-positive bacteria, Mycobacterium tuberculosis, Enterobacteriaceae, Pseudomonas and Neisseria gonorrhoeae has become more complex. The annual identification of numerous occurrences of infections resistant to multiple drugs underscores a burgeoning global health crisis. This trend results in extended hospitalizations, the requirement for costlier antibiotic treatments, and an upswing in rates of illness and death from bacterial diseases. Furthermore, the rise of 'superbugs' is a profound risk to public health, underscoring the urgency of addressing the issue of drug resistance.About 40 to 80 percent of bacteria on Earth are able to form biofilms, communities of bacteria wrapped in a homegrown polymer matrix of polysaccharides, proteins, and extracellular DNA. In humans, biofilms are commonly found on skin, mucous membranes, and tooth surfaces. Chronic wounds, such as diabetic foot ulcers, stress injuries, and venous ulcers of the lower extremities, are serious problems worldwide, and these wounds are difficult to heal, in part because of the disturbed physiological processes and wound environment that are significantly influenced by a variety of microorganisms [3].The complex interactions between these microorganisms lead to the infection mechanisms associated with biofilms. Bacterial biofilms are not only resistant to antibiotics, but also show resistance to ultraviolet rays, light metals and heavy metals. Micronutrients and wound exudates, which aid wound recovery, instead become hotbeds for a variety of microorganisms to form biofilms. It is estimated that biofilms are present in about 60 % of chronic wounds [4]. Studies have shown that about 80 % of bacterial infections involve the formation of bacterial biofilms, and the bacteria in these biofilms differ significantly in morphology and physiological function from free-living bacteria. They can be 10 to 1000 times more resistant to antibiotics and can effectively resist the host's immune defense mechanisms, becoming a major factor in the problem of bacterial resistance [5].Bacterial biofilms in the mouth are a major contributor to the increase in antimicrobial resistance, and they can trigger serious diseases including periodontal and gum disease. Ullah et al. designed a nanocomposite based on essential oils. The results show that there is good compatibility between the main active ingredients in essential oils. In addition, the combination of cinnamon and clove EOs showed significant antibacterial, antiquorum and anti-biofilm effects [6]. Bacterial biofilms can cause persistent infectious diseases and pose a huge obstacle to anti-infective treatment. The unique structure and composition of bacterial biofilms limit the penetration ability of drugs and weaken the efficacy of antibiotics, thus exacerbating the harm caused by bacterial biofilms [7].Using nanotechnology to solve the problem of bacterial biofilm has become a promising strategy [8].Bacteria can sense and respond to environmental signals that promote exopolysaccharide (EPS) synthesis. Therefore, if the synthesis of EPS can be inhibited or blocked, the formation of biofilm can be limited [9,10]. Bacterial biofilms can survive harsh environments and are resistant to conventional antibiotic treatment compared to traditional free bacteria. The combined action of NO gas and type I PDT not only enhances the ability of PS to penetrate the EPS barrier, but also enables PDT to effectively eliminate bacterial biofilms in anoxic environments. Lu et al. prepared MSN@MOF/SNP NPs (MMS NPs) for synergistic PDT and NO treatment of biofilm infections. Antibacterial experiments of in vitro and in vivo biofilms demonstrated the remarkable effect of MMS NPs on removing bacterial biofilms under laser irradiation [11].

### 1.2 Emerging antimicrobial strategies

Misuse of traditional antibiotics has hastened the evolution of resistance to multi-drugs, underscoring an immediate requirement for creating potent and reliable alternatives to combat microbial threats. Cationic bioactive polypeptides, known as antimicrobial peptides (AMPs), exhibit lethal action against a broad spectrum of bacteria, including both Gram-positive and Gram-negative strains [12]. These peptides interact with the anionic phospholipids of bacterial membranes through electrostatic forces, resulting in membrane perturbation or disintegration, and consequently, the demise of the bacteria. A key distinction between AMPs and conventional antibiotics is that AMPs do not target specific enzymatic sites, which significantly reduces the likelihood of resistance development. Despite their promise, the clinical deployment of AMPs faces several challenges. These include the high costs associated with production, potential toxicity issues, the need for high dosages, and susceptibility to the biological milieu, such as susceptibility to protease degradation and lack of stability at high physiological salt concentrations [13]. Addressing these limitations is crucial for the successful translation of AMPs into effective clinical treatments. Throughout history, metals and metal ions have served as conventional antimicrobial substances, with metal ions like Ag^+^, Cu^2+^, Hg^2+^, and As^3+^ being toxic to most bacteria [14]. Most metal-based antimicrobial agents are believed to exert their antimicrobial effects by forming metal-biomolecule complexes, primarily through three pathways: binding of metal ions to functional groups in biomolecules, replacement of central metal ions in enzymes, and binding of metal ions to thiol groups in enzymes. These processes disrupt enzymes, cell membranes, and deoxyribonucleic acid (DNA) structures, leading to abnormal bacterial physiological activities and death. While metal-derived antimicrobial substances are effective in killing bacteria, concerns about their possible harmful effects on human health restrict their application in clinical setting. Cationic polymers, such as quaternary ammonium, imidazolium, chitosan, and ε-poly-L-lysine, exhibit good antimicrobial effects. Their antimicrobial mechanisms include the following processes: the positively charged component of the antimicrobial peptide binds to the phospholipid molecules on the bacterial membrane through electrostatic interactions, while the hydrophobic chain segment inserts and penetrates the hydrophobic region, causing the leakage of cytoplasmic contents, including DNA and ribonucleic acid (RNA), leading to bacterial death. Currently, research on cationic polymer antimicrobial agents is mainly confined to the laboratory, and their biocompatibility requires further investigation. In recent years, other therapeutic methods such as gas therapy, PDT, PTT, and mild photothermal therapy (MPPT) have been applied in anti-infection treatment. In the last ten years, gas therapy has been a focus. H_2_, H_2_S, SO_2_, and NO serve dual functions: they act as crucial messengers within the body and significantly influence a variety of disease mechanisms. These gases, capable of killing bacteria and dispersing biofilms, are of interest as antimicrobial agents due to their potential to promote wound healing caused by bacterial infections without inducing bacterial resistance [15].

### 1.3 Phototherapy in antimicrobial therapy

Phototherapy is a method of treating diseases using light of specific wavelengths. It leverages the therapeutic properties of light to target and treat various conditions. Phototherapy has emerged as a leading therapeutic approach for combating bacterial infections, distinguished by its rapid action, low side effects, and the lack of associated drug resistance issues. Near-infrared (NIR) light possesses distinctive advantages in phototherapy, particularly in high signal-to-noise ratio and deep penetration. The penetration depth of NIR light can reach 5–10 mm, significantly exceeding the penetration capability of visible light, which is typically in the range of 1–2 mm. Additionally, biological tissues exhibit minimal absorption of NIR light, and the tissues' scattering coefficient of the tissues is relatively low, reducing the impact on superficial tissues. When biological tissues are exposed to light, they may exhibit autofluorescence. Due to its longer wavelength, NIR light is less absorbed by tissue autofluorescence, thereby providing a higher signal-to-noise ratio. This implies that under NIR light, the contrast between signals, such as the fluorescence of biological markers, and background noise is enhanced, facilitating more accurate detection and imaging. The two most common types of phototherapy include PDT and PTT. PDT leverages photosensitizing agents to initiate the formation of singlet oxygen (^1^O_2_) when exposed to light, leading to the demise of microbes. Conversely, PTT entails the transformation of light energy into thermal energy by photosensitizers, resulting in localized hyperthermia that inactivates bacteria. Given that both modalities are typically conducted within the near-infrared range (650–900 nm), their synergistic application presents a potent strategy for enhancing antibacterial efficacy.

PTT utilizes photothermal agents (PTAs) to transform absorbed light into thermal energy, leading to elevated temperatures capable of inducing protein denaturation and thermal destruction of bacteria. Beyond its rapid bacterial structural and functional disruption, the heat generated from light also fortifies the immune system's response to combat intracellular bacterial infections. PTT has become a promising treatment modality for multidrug-resistant (MDR) pathogens, presenting as a credible alternative to antibiotics. This is due to several benefits: its wide-ranging antibacterial impact on both Gram-positive and bacteria Gram-negative, regardless of their membrane types, thanks to the penetrating properties of light sources such as NIR; its capacity for deep tissue penetration without tissue harm, facilitated by the NIR light source within the 700–1400 nm range, thereby increasing the likelihood of effective bacterial eradication in deep-seated tissues; its application of localized hyperthermia to target bacteria while minimizing harm to surrounding healthy cells; its enhancement of antibacterial agent diffusion into biofilms through induced hyperthermia; and its non-invasive, contact-free mechanism that reduces the likelihood of bacterial resistance development [[16], [17], [18]]. The efficacy of PTT is contingent on the specific PTAs employed, with ongoing research into multiple generations of PTAs for their applicability in antibacterial settings. Among these, photothermally active nanoparticles (PANs) have emerged as a leading class of antimicrobial PTAs within the domain of antibacterial research.

PDT employs the interaction between photosensitizers with specific wavelengths of light to produce cytotoxic substances, such as singlet oxygen, to eradicate target bacteria or cells. The process involves the activation of the photosensitizer, where these organic molecules, either natural or synthetic, absorb light energy at specific wavelengths and are excited to a higher energy state. Subsequently, ROS was induced as a result of the excited state photosensitizer interacting with nearby oxygen and water molecules, resulting in the creation of highly reactive and cytotoxic agents such as ^1^O_2_, H_2_O_2_, and ·OH. These ROS inflict damage on bacteria through multiple mechanisms: they can cause the bacterial cell membrane to be compromised, resulting in the escape of intracellular substances and a reduction in membrane integrity; they can oxidize proteins and enzymes within the bacterial cells, thereby impeding the metabolic processes and growth of the bacteria; they can also damage bacterial DNA, hindering replication and transcription, thus inhibiting bacterial reproduction. Ultimately, under the influence of ROS, bacterial cells undergo programmed cell death (apoptosis) or unregulated cell death (necrosis). Due to its high selectivity and localized action, PDT has a minimal impact on normal tissues, making it a promising antibacterial treatment method, especially in cases where traditional antibiotics are ineffective or have led to drug resistance. The benefits of PDT include: (1) a wide-ranging efficacy due to the generation of ROS that interfere with various bacterial metabolic pathways and cellular structures, not just a single target; (2) the antibacterial effect is triggered by light irradiation, utilizing materials that produce ROS upon light exposure, with a very low likelihood of bacterial resistance developing to these materials; (3) selective targeting of bacteria at the site of infection, with precise control over the light application's timing and area; (4) minimal toxicity to healthy cells during treatment of bacterial infections in living organisms; (5) compatibility with other treatment modalities, including radiotherapy, chemotherapy, and photothermal therapy; and (6) a low likelihood of resistance traits being rapidly passed on to subsequent bacterial generations. Consequently, the potential for bacterial resistance to PDT is significantly diminished.

### 1.4 Advantages of nanomaterials in antimicrobial applications by PTT and/or PDT effect

**Fig. 1:**
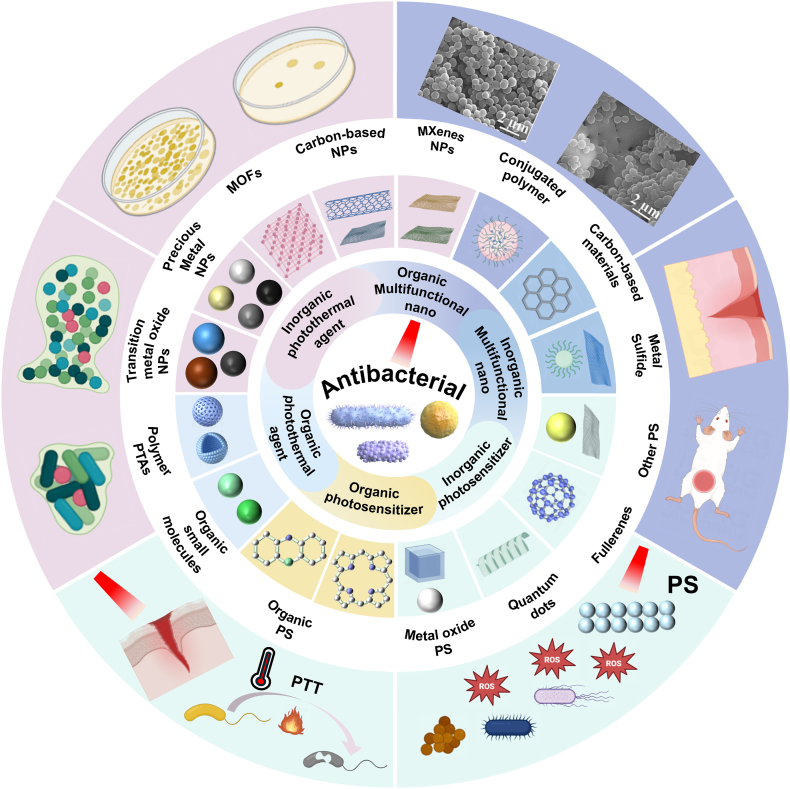
Schematic diagram of nanomaterials for antimicrobial therapy.

Nanomaterials, serving as photosensitizers in the field of antimicrobial therapy, have demonstrated significant advantages that greatly enhance therapeutic efficacy and expand the scope of antimicrobial treatment applications. The benefits of nanomaterials within the realm of antimicrobial phototherapy stem are reflected in their unique physicochemical properties and therapeutic mechanisms. The photothermal phenomenon involves materials capturing light of specific wavelengths and transforming this radiant energy into thermal energy. Owing to their elevated surface-area-to-volume ratios and adjustable optical properties, nanomaterials possess the capability to effectively engross light energy and swiftly transmute it into warmth, exerting a thermal effect on bacteria [19,20]. This thermal effect can generate local high temperatures around the nanomaterials, quickly killing or inhibiting bacterial growth with minimal impact on surrounding normal tissues. The high photothermal conversion efficiency of nanomaterials allows for sufficient heat generation under low laser power, reducing thermal damage to surrounding tissues. Moreover, the dimensions and contour of nanomaterials can be meticulously tailored to optimize their photothermal performance and biocompatibility. For instance, gold nanorods have become a research hotspot renowned for their exceptional ability to transform light energy into heat and good biocompatibility. They can produce a strong photothermal effect under near-infrared light irradiation, effectively killing a variety of bacteria. Nanomaterials as photosensitizers in PDT also show significant advantages in the antimicrobial field. PDT is a non-thermal effect approach that uses the combined action of photosensitizers, oxygen and light to produce photochemical effects to kill bacteria. As widely used photosensitizers, nanomaterials can excite ROS when exposed to light. These highly reactive oxygen molecules can react with cellular biomolecules within bacteria, causing the destruction of cellular structure and function, thereby achieving antimicrobial effects [21]. The production process of green nanomaterials does not use or minimizes the use of harmful chemicals. Such materials usually have good biocompatibility, reducing potential harm to the human body and the ecosystem. İpek et al. adopted a simple and environmentally friendly approach to prepare Au NPs with environmental friendliness, simplicity, economy and high efficiency. The results showed that Staphylococcus aureus and Candida albicans showed the highest antibacterial activity against 0.06 mg/ml Au NPs [22]. The nanoparticles synthesized by this green technology demonstrate the feasibility of green nanomaterials for antimicrobial use. Xu et al. studied the preparation of non-toxic, stable and small-sized silver nanoparticles from potato (Solanum tuberosum, ST) peel extract using its own conditions by green synthesis technology. It is worth noting that by comparing the antibacterial effect of standard antibiotics, silver nitrate solution and ST-Ag NPs, it was found that ST-Ag NPs showed a more significant inhibitory effect on pathogenic microorganisms at lower concentrations [23]. Nanomaterials can also be modified on their surface to enhance their targeting ability, selectively binding and killing specific bacteria by connecting specific ligands or antibodies, improving therapeutic efficacy while reducing the impact on normal cells. In addition, the multifunctionality of nanomaterials allows them to carry multiple drugs simultaneously, combining with other treatment methods such as chemotherapy and immunotherapy, to achieve multimodal synergistic therapy. This synergistic effect can significantly improve antimicrobial effects, reduce treatment time and drug dosage, and decrease side effects. PTAs play a key role in photothermal therapy of PTT. The selection and application of suitable PTAs is crucial to the successful implementation of PTT [24]. With the rapid progress of nanotechnology, many types of inorganic nanomaterials (such as metals, metal oxides, metal sulfides and carbon-based nanomaterials) and organic nanomaterials (such as small molecule polymers, conjugated polymers) have been widely studied and developed. Similarly, PS, as a PDT medium, plays a key role in the production of ROS using light and oxygen. Many types of inorganic nano PSs (such as transition metal oxides, quantum dots etc.), organic nano PSs (such as Porphyrin, Phenothiazine etc.), have been extensively studied (see Fig. 1).

## 2 Photosensitizers for antibacterial PTT or PDT

### 2.1 Photosensitizers for antibacterial PTT

#### 2.1.1 Organic nano photothermal agents

Organic photosensitizers have demonstrated a range of advantages in photothermal and photodynamic therapy, while also facing some challenges. Their advantages include a high degree of designability, enabling the adjustment of molecular structures through chemical synthesis to optimize photophysical properties and biocompatibility, achieving precise control over the light absorption range, fluorescence characteristics, photostability, and ROS generation efficiency. This designability allows organic photosensitizers to be tailored to specific therapeutic needs, adapting to different types of lesions and treatment environments. Moreover, organic photosensitizers typically exhibit good biocompatibility and low toxicity, which helps to reduce side effects during treatment. They also possess excellent photothermal conversion efficiency and photochemical activity, capable of rapidly generating heat or ROS under light irradiation for effective treatment of pathological tissues. Certainly, the implementation of organic photosensitizers in practice also faces limitations. For instance, their photostability may be insufficient, prone to degradation under multiple or prolonged light exposures, which can affect therapeutic outcomes. Additionally, organic molecules may face challenges in metabolism and clearance within the body, requiring further optimization to enhance in vivo stability and reduce potential long-term toxicity. Some organic photosensitizers have poor water solubility, which may necessitate additional surface modifications or carrier systems to improve their solubility and distribution uniformity within biological systems. Furthermore, the photothermal and photodynamic effects of photosensitizers can be constrained by the extent to which light penetrates tissues, thus requiring the selection of appropriate light sources and irradiation parameters to ensure effective treatment.

#### 2.1.2 Organic small molecules

**Fig. 2:**
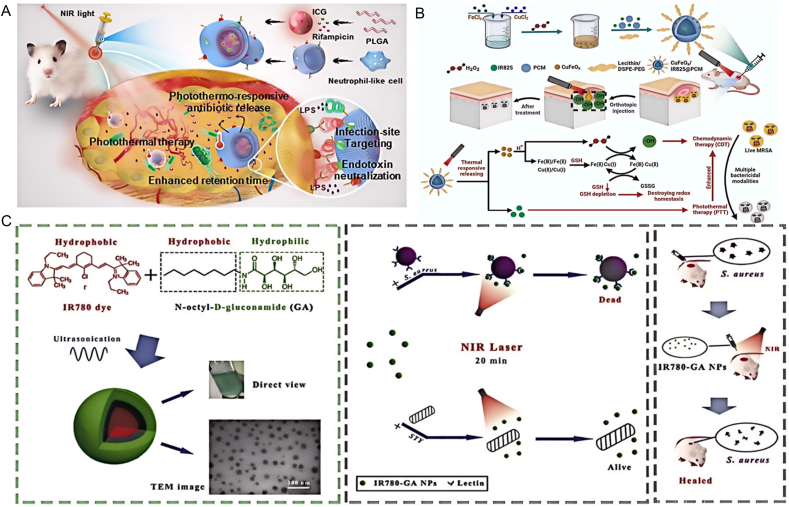
Antibacterial application of organic small molecule photothermal agents. (A) A schematic illustration of a photothermal drug delivery system coated with a neutrophil-like cell membrane for the combined treatment of *P. aeruginosa* infections. Reproduced with permission from Ref. [28]. (B) A schematic portrayal of the creation of CuFeO/IR825@PCM and its dual mechanism of PTT and CDT for combating bacterial infections. Reproduced with permission from Ref. [29]. Copyright 2024,Elsevier B.V. (C) Schematic representation for the preparation of IR780-GA and the mechanism of PTT for antibacterial anti-infective treatment. Reproduced with permission from Ref. [30]. Copyright 2023,Elsevier B.V.

Organic small molecules, defined by their minimal molecular weight and simple structure, act as potent photothermal agents capable of triggering photothermal antibacterial effects and aiding in wound recovery [25]. These molecules exhibit high light absorption capabilities and a superior photothermal conversion rate, enabling them to generate substantial heat with minimal light exposure and power, thereby yielding potent therapeutic outcomes. Organic small molecules, including cyanine dyes (indocyanine green (ICG), tryptophan (Cytate),and IR 780 and IR 825), benzo[1,2-c:4,5-c′]bis ([1,2,5]thiadiazole)BBTD, 4,4-difluoro-boradiazaindacene (BODIPY), have excellent biocompatibility and biodegradability, and have great prospects in clinical antibacterial PTT. ICG possesses excellent biocompatibility, and its near-infrared light excitation wavelength can penetrate deeper into the tissue, producing relatively higher heat [26]. Topaloglu et al. irradiated ICG with 808 nm laser and it exhibited a 95 % antibacterial efficacy against both Gram-positive and drug-resistant strains [27]. Indeed, the US FDA has granted clearance for ICG's use in medical imaging and for addressing internal contamination due to cesium or thallium. During a clinical study, ICG was utilized in PTT to combat periodontitis, demonstrating a reduced likelihood of promoting bacterial resistance and exhibiting less toxicity to oral cells than chlorhexidine gel. Furthermore, Xu and colleagues developed ICG/LUT-CS nanocomposites, which, when activated by an 808 nm laser, displayed substantial thermal therapeutic effects and compromised the structural integrity of bacteria (Fig. 2A) [28]. It also showed obvious elimination of S.aureus biofilm and cell morphology deformation, suggesting a synergistic effect between PTT and chemotherapy in enhancing bacterial killing efficiency. Xu et al. prepared hydrophilic bacteria-targeted photothermal nanoparticles IR780-GA NPs with a PCE of 55.8 %. In vivo antibacterial experiments indicated that IR780-GA NP with NIR could eliminate most of the bacteria at the infection site (Fig. 2B) [29]. Photothermal therapy induced by medium concentration IR780-GA NP had a stronger effect on microbial inactivation of MRSA than vancomycin. Su et al. prepared the NIR photoresponsive TD coating microneedle patch, which was loaded with W379 and IR780, and found that the temperature of the microneedle patch mixed with 0.4 wt% IR780 rapidly increased to 47.8 °C within 180 s (Fig. 2C) [30]. Upon applying the patch to a bacterial suspension and immersing the microneedle array for 2 h, subsequent exposed to NIR for varying durations, an ample amount of the antimicrobial peptide W379 was released from the microneedle patch. This was enough to nearly eradicate all MRSA bacterial in the suspension on a PDMS surface following irradiation for 5 min or longer. These advancements highlight the potential of organic small molecules in revolutionizing antibacterial treatments. BBTD molecules form a highly distorted molecular configuration due to the steric hindrance effect between their highly electrodeficient receptors, which enables them to change from aggregation-induced fluorescence (ACQ) to aggregation-induced luminescence (AIE) effect, thereby improving AIE activity. This property allows BBTD to maintain a large dihedral Angle even in the aggregation state, reducing the intermolecular π-π stacking, improving the luminous efficiency, and further enhancing its photothermal conversion capability [31]. Li et al. synthesized a photothermal molecule TPE-BT-BBTD, encapsulated the TPE-BT-BBTD molecule in polylactic-glycolic acid (PLGA) core and coated with cell membrane to form BBTD@PM NPs. BBTD@PM NPs can reach about 70 °C within 10 min after laser irradiation, showing excellent photothermal effect and can achieve photothermal inactivation of pathogenic microorganisms. At the same time, after several heating and cooling cycles induced by 1064 nm pulsed laser, their photothermal conversion efficiency remained stable. In addition, BBTD@PM NPs only binds to bacteria of the genus Mycobacterium, but not to other types of bacteria, indicating that they have specific targeting capabilities. In vivo antibacterial experiment showed that under 1064 nm laser irradiation, the bactericidal effect of BBTD@PM NPs group was significantly superior to other treatment groups, and even significantly exceeded the combined effect of first-line antibiotics [32]. BODIPY dyes have advantages such as excellent brightness and quantum yield, and through in-depth study of the photophysical and photothermal properties of a series of π-extended BODIPY dyes with high absorption in the near infrared region, excellent photothermal conversion efficiency of more than 90 % has been successfully achieved [33]. Song et al. prepared BBDH NPs based on a BODIPY probe, a NO thermal response donor, and a pegylated polymer. At 685 nm irradiation (1.0 W cm^−2^, 10 min), the temperature of the BBDH NPs solution increased from 25.0 °C to 56.7 °C, indicating its significant photothermal effect. In vitro antimicrobial tests showed that compared with BDH NPs + laser treatment group, BBDH NPs + laser treatment group showed a higher bactericidal rate and more significant therapeutic effect. In addition, on day 12, compared with the control group, the wound healing of the mice treated with BBDH NPs combined with laser irradiation was significantly improved, and the wound area was significantly reduced [34].

##### 2.1.2.1 Polymer photothermal agent

**Fig. 3:**
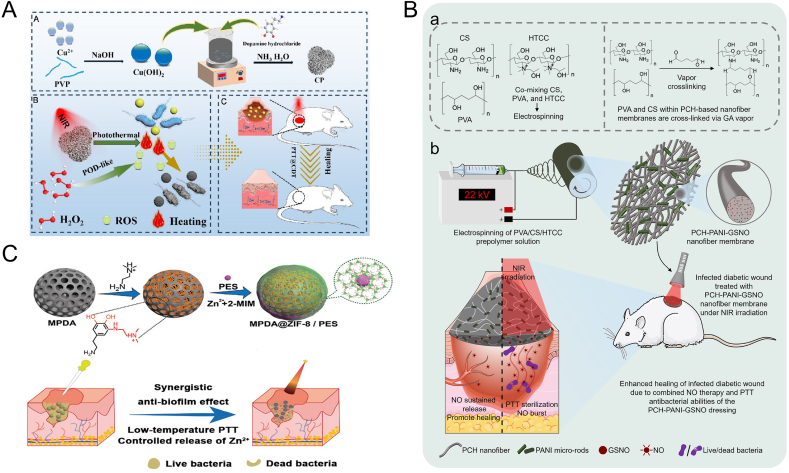
Antibacterial application of polymer photothermal agent.(A) A schematic illustration of the wound healing process mediated by CP nanozymes through photothermal action. Reproduced with permission from Ref. [36]. Copyright 2024,American Chemical Society. (B) (a) Schematic diagram of cross-linking between PVA and CS in PCH electrospinning nanofibers using GA steam.(b) The process of PCH-PANI-GSNO nanofiber membrane helping to heal infectious DW was demonstrated. Reproduced with permission from Ref. [43]. (C) A diagram showing the elimination of biofilm through the regulated discharge of Zn^2+^ ions and mild PTT. Reproduced with permission from Ref. [37]. Copyright 2021,The Royal Society of Chemistry.

Polymeric nanomaterials are gaining prominence in PTT, attributed to their exceptional biocompatibility, efficient photothermal conversion, and minimal in vivo toxicity. These characteristics position them as leading contenders for advanced therapeutic applications. Notably, polymers likepoly(3,4-ethylenedioxythiophene) (PEDOT), polyaniline (PANI), and polydopamine (PDA) have emerged as a distinguished group of photothermal agents. They are prized for their superior absorption capabilities, robust photothermal stability, high conversion efficiency, and the economic advantage of low-cost production. In particular, PDA has garnered significant interest for its use in magnetically targeted photothermal therapy (MPTT). Its high PCE, combined with its remarkable biocompatibility, hydrophilicity, and adaptability to various modifications, makes it an ideal candidate for MPTT. Polydopamine, produced through the self-assembly of dopamine under mildly basic conditions, mimics the strong adhesiveness of mussels, providing a simple and direct method of synthesis. It boasts an array of superior attributes, including straightforward preparation, outstanding biocompatibility, and distinguished photothermal performance, solidifying its role as a cutting-edge material in the realm of PTT. The PDA structure is replete with catechol moieties and primary and secondary amine groups, endowing it with the unique ability to adhere to a vast array of solid substrates, thereby forming a thin, adherent PDA film. This film not only serves as a robust coating but also acts as a multifunctional "intermediary" for surface modification. The nucleophilic sites on the PDA film enables it to undergo reactions such as Schiff base formation or Michael addition with a variety of reagents, facilitating the introduction of diverse functional groups onto material surfaces. Leveraging its intrinsic properties, PDA has emerged as a promising antibacterial material. Its proficient photothermal conversion capability, coupled with the abundance of catechol and amine structures, endows PDA with potent antibacterial activity. The hydrogen peroxide generated by PDA can induce protein denaturation on bacterial cell membranes, leading to structural disruption and ultimately, bacterial demise. This mechanism positions PDA as an innovative and highly effective biocidal agent. Furthermore, PDA's rich chemical reactivity, combined with its mild synthesis strategies, has broadened its applications in the interfacial chemical modification of antibacterial composite materials. Its versatility in surface functionalization allows for the creation of tailored antibacterial surfaces, enhancing the efficacy and scope of antimicrobial strategies. Xu and colleagues crafted a dual-functional hybrid system composed of gold nanoparticles and PDA-assisted by HAp. The nanoparticles not only promoted the expression of key genes (COL III, COL I, bFGF) in NIH3T3 cells, subsequently aiding in the development of granulation tissue and the generation of collagen, thus speeding up the wound healing process [35]. At a photoinduced temperature of 45 °C, the nanoparticles exhibited a high antibacterial efficacy of 96.8 % against Escherichia coli and 95.2 % against S.aureus, without causing damage to surrounding healthy tissues. The material's bactericidal efficiency surpassed that of processes catalyzed by peroxidase or photothermal therapy alone. Luo et al. has recently achieved a groundbreaking scientific result by successfully fabricating a novel type of nanoparticle—Cu_x_O@PDA (referred to as CP). This nanoparticle not only exhibits exceptional photocatalytic efficiency but also possesses peroxidase-like activity, capable of generating hydroxyl radicals (•OH) during the conversion of hydrogen gas (H_2_) to hydrogen peroxide (H_2_O_2_), a process that is particularly efficient under near-infrared (NIR) irradiation.Excitingly, a synergistic effect exists between the peroxidase-like activity and photothermal activity of CP nanoparticles, which significantly enhances their antibacterial capabilities. In experiments, this new type of nanoparticle has demonstrated astonishing antibacterial effects, capable of eradicating up to 99.74 % of Staphylococcus aureus (*S. aureus*) and 99.82 % of Escherichia coli (*E. coli*), both of which are common infectious pathogens. Furthermore, the research team conducted in vivo treatment experiments, which indicated that the excellent antibacterial performance of CP nanoparticles is equally remarkable in the treatment of actual wound infections(Fig. 3A) [36]. Peng et al. developed mesoporous polydopamine (MPDA) core-shell nanoparticles coated with ZIF-8, which upon near-infrared irradiation, elevated the temperature at the infection site [37]. The ZIF-8 shell loaded with Pifithrin-μ (PES) facilitated the release of PES, which diminished the bacteria's heat resistance within the biofilm. The liberated PES mitigated the bacteria's thermal resistance within the biofilm, enabling effective low-temperature PTT for biofilm infections (Fig. 3C). It was worth noting that the photothermal conversion capacity of PDA destroyed the biofilm matrix, which might be another mechanism for PDA to exert antibacterial activity. Utilizing various monochromatic continuous-wave lasers, PDA is capable of efficiently transforming light energy into thermal energy, with a photothermal conversion rate of approximately 45 % in the infrared spectrum [38]. Furthermore, PDA can be integrated with other substances to fabricate a more stable and adherent antimicrobial wound dressing. Guo and colleagues created a Mg^2+^ enriched PDA-polyacrylamide composite hydrogel for the treatment of infected wounds, which, under NIR irradiation, achieved a kill rate exceeding 95 % against *E. coli* and *S. aureus* [39]. Huang and team engineered a multifunctional PDA-modified mesoporous nanoparticle-curcumin-loaded hydrogel for wound infection treatment [40]. This hydrogel was able to elevate the temperature at the wound site and regulate the release of curcumin, effectively curbing bacterial infections and demonstrating outstanding biocompatibility. The modification of PDA with metal ions, such as Ag ^+^ by Qi et al. and Cu^2+^ by Li et al., has been shown to enhance its photothermal conversion efficiency and antimicrobial potency, contributing to the advancement of photothermal-assisted wound healing [41,42]. Xie et al. have successfully developed a novel multifunctional wound dressing membrane by integrating polyaniline (PANI) and S-nitrosoglutathione (GSNO) into a matrix of polyvinyl alcohol, chitosan, and hydroxypropyl trimethyl chitosan chloride (PVA/CS/HTCC), prepared using electrospinning technology. This state-of-the-art dressing membrane combines the dual advantages of photothermal antimicrobial therapy and nitric oxide gas therapy, demonstrating exceptional persistent bactericidal effects and biofilm disruption capabilities against a variety of bacteria, including methicillin-sensitive Staphylococcus aureus, methicillin-resistant Staphylococcus aureus, and Escherichia coli. Particularly noteworthy is the significant synergistic activation between the photothermal effect (PTT) and nitric oxide release of the dressing membrane. Under near-infrared (NIR) irradiation, the membrane is capable of simulating a nano-fuse mechanism, achieving an explosive release of nitric oxide, effectively decomposing biofilms. Furthermore, the uniform release of nitric oxide from the nanofibers promotes angiogenesis and cell migration, which are crucial for wound healing. In experiments conducted on a diabetic wound model in rats, this dressing membrane significantly facilitated the wound healing process within a short period of 14 days(Fig. 3B) [43]. PEDOT nanoparticles have broad absorbance in the near infrared band (700–1250 nm), positioning them as photothermal coupling agents in the near infrared band. In research by Ko et al., a novel photothermal nanocomposite hydrogel was created by combining agarose with PEDOT nanoparticles that were adorned with poly(styrene-sulfonate) (PEDOT:PSS) [44]. The ICG on the PEDOT surface enabled the production of ROS under NIR light for PDT, and the PEDOT itself showed substantial absorption in the NIR II range, leveraging its inherent photothermal effects for PTT. The synthesized PEDOT:ICG@PEG-GTA showcased a high photothermal conversion efficiency of 71.1 %, superior photostability, minimal toxicity, and remarkable bactericidal activity of nearly 99 % across both the NIR I and II windows. These findings on PEDOT:ICG@PEG-GTA signify a novel therapeutic approach for combating bacterial infections and highlight its potential as an outstanding agent in nanomedicine-based phototherapy [45]. Hydrogels leveraging PANI as a photothermal component are anticipated to evolve into innovative wound dressings, designed to manage bacterial infections and enhance the healing process of skin wounds. In addition to its superior photothermal conversion capability, PANI possesses unique photostability, enabling it to achieve the effect of repeated photothermal therapy, which can effectively inhibit bacterial proliferation, promote angiogenesis, and encourage cell multiplication. The integration of PANI with other biomaterial components allows for the creation of hydrogels with improved biocompatibility and mechanical integrity [46]. In a study by Pang and colleagues, hydrophobic PANI was covalently attached to hydrophilic chains of ethylene glycol methacrylate chitosan (CS) to produce PANI nanoparticles with double-bond termination [47]. These nanoparticles were adept at converting NIR radiation into heat for bactericidal purposes, unrestricted by temporal or spatial constraints. Moreover, the nanoparticles bolstered the hydrogel's mechanical properties and curtailed the spread of the photothermal antimicrobial agents to adjacent tissues. Kim et al. synthesized polyvinylpyrrolidone sulfobetaine (PVPS) by reacting quaternized polyvinylpyrrolidone with 4-hydroxyphenylacetone (C-PVP) and 1,3-propanesultone. Subsequently, PANI was modified with PVPS to create an antibacterial coating based on PANI (PVPS:PANI). The coating (1 mg/mL) could effectively eradicate both Gram-positive and Gram-negative bacteria after exposure to NIR for 3 min [48]. Zhou et al. utilized a hydrothermal synthesis to produce uniformly structured PANI nanomaterials, enhanced with F127 modification (referred to as F-PANPs). These nanomaterials demonstrated favorable attributes, including optimal size, robust NIR light absorption, and an elevated PCE reaching 48.5 %, along with potent therapeutic capabilities [49].

#### 2.1.3 Inorganic photothermal agent

Compared with organic nanomaterials, the greatest advantage of inorganic nanomaterials as photosensitizers is their superior photothermal conversion rates. They are capable of efficiently transforming absorbed light energy into thermal energy during photothermal therapy, so as to generate enough heat to eliminate the diseased tissue in the treatment process. This efficient conversion ability, combined with their chemical and thermal stability, provides an effective solution for photothermal therapy of deep tissues. In addition, the versatility of inorganic nanomaterials also allows them to achieve a variety of therapeutic functions in a single system, further improving the efficiency and effect of treatment.

##### 2.1.3.1 Precious metal nanomaterials

Noble metal-based PTAs, such as gold (Au), silver (Ag), palladium (Pd), platinum (Pt), and ruthenium (Ru), are distinguished by their exceptional photophysical characteristics. Typically recognized for their chemical inertness, these materials display pronounced surface plasmon resonance (SPR), substantial light absorption cross-sections, adjustable aspect ratios, and elevated PCE within NIR spectrum. He et al. fabricated ultrathin two-dimensional AuPd alloy nanosheets, approximately 1.5 nm in thickness, leveraging the properties of these noble metals [50]. These nanosheets displayed a high PCE (η = 76.6 %), stable optical signal, and potent ROS. Upon exposure to NIR, the AuPd nanosheets were capable of completely eradicating both S.aureus and *E. coli*. Upon the absorption of light at wavelengths corresponding to their absorption spectra, the free electrons in noble metal nanoparticles vibrate along the surface of the particles. This distinctive photophysical phenomenon, termed LSPR, facilitates the photothermal conversion of noble metal nanomaterials, rendering them highly effective for photothermal antimicrobial therapies [51,52]. The location of the LSPR band in the NIR spectrum and the resulting heat production are regulated by the surface charge density, which is affected by variables including the structure, shape, size, dielectric constants, form, and interactions between the noble metal nanoparticles.

**Fig. 4:**
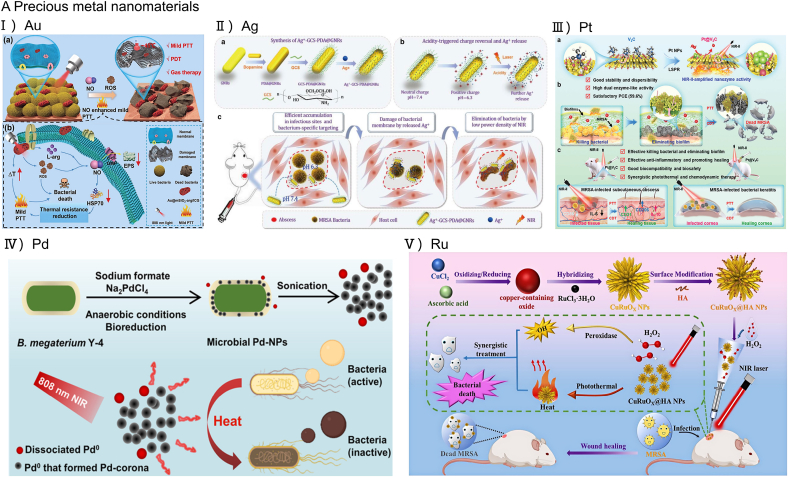

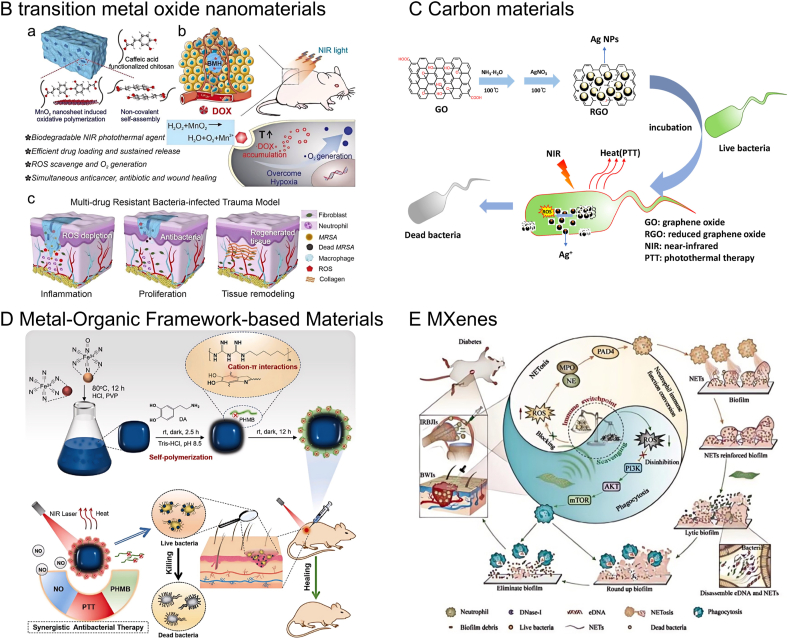
Antibacterial application of inorganic photothermal agent.(A).Precious metal nanomaterials used for performing PTT. (A-Ⅰ) Schematic presentation of antibacterial mechanisms of Au@mSiO_2_-arg/ICG NPs Reproduced with permission from Ref. [55]. Copyright 2024,Wiley-VCH GmbH. (A-Ⅱ) A diagram of the fabrication of Ag^+^-GCS-PDA@GNRs for the bacteria-specific targeting and the integrated chemo-photothermal therapy. Reproduced with permission from Ref. [66]. (A-Ⅲ) Schematic illustration of the construction, antibacterial, and anti-infective therapy of Pt@V_2_C nanoplatforms with photothermal and chemo dynamic therapy. Reproduced with permission from Ref. [70]. Copyright 2024,Wiley-VCH GmbH. (A-Ⅳ) Schematic representation for the preparation of Pd NPs and the mechanism of PTT for antibacterial treatment. Reproduced with permission from Ref. [75]. (A-Ⅴ) A diagram illustrating the fabrication of CuRuOX@HA NPs and the operational mechanism during PTT/CDT in addressing infections caused by multi-drug-resistant bacteria. Reproduced with permission from Ref. [76]. Copyright 2023,The Royal Society of Chemistry. (B) A diagrammatic depiction of the BMH hydrogel for effective treatment of melanoma and wound healing in MRSA infections. Reproduced with permission from Ref. [83]. Copyright 2020,Elsevier Ltd. (C) Schematic diagram of synergistic antibacterial action of RGO/Ag nanocomposites under near-infrared radiation. Reproduced with permission from Ref. [100]. Copyright 2020,Elsevier B.V. (D) A diagrammatic depiction of the creation of PB-NO@PDA-PHMB and its combined therapeutic approach for the healing of wound infection. Reproduced with permission from Ref. [101]. Copyright 2023,Elsevier B.V. (E) Schematic describing the mechanism of DNase-I@V_2_C in combating diabetic related biofilm infections. Reproduced with permission from Ref. [99].

Gold is known for its chemical stability, high malleability, and versatility in terms of its physical attributes such as shape, morphology, size, and structure. It is also highly resistant to corrosion and oxidation. In PTT, gold nanomaterials have become a focal point due to their superior light absorption, stability, tunable optical characteristics, and efficient heat generation attributed to the presence of a large number of free electrons [53]. A range of gold nanostructures with effective NIR light absorption have been developed, encompassing nanoshells, nanocages, nanospheres, nanostars and nanorods. Au nanoparticles exhibit a high infrared light absorption rate and a low photoluminescence quantum yield, enabling efficient photon-to-phonon conversion, making it one of the candidate materials for an efficient photothermal agent [54]. Zhou et al. developed an integrated phototherapy and gas therapy platform based on Au@mSiO_2_-arg/ICG NPs [55]. Specifically, the nanoparticles exhibited superior photothermal performance under 808 nm light exposure and also functioned as ROS producers, subsequently catalyzing the conversion of l-arginine to release nitric oxide gas (Fig. 4A-Ⅰ).Kuo et al. performed experimental analyses to assess the potency of gold nanoparticles as antibacterial agents when complexed with a range of antibiotics. The findings demonstrated that gold nanoparticle-antibiotic combinations had increased effectiveness in inhibiting growth and eradicating both Gram-positive and Gram-negative bacteria compared to the antibiotics used alone [56]. Similarity, Wang et al. utilized amino polyethylene glycol thiol (NH_2_-PEG-SH) to thiolate vancomycin, thereby conveniently anchoring it onto gold nanostar materials (AuNSs). They developed a vancomycin-decorated gold nanostar material (AuNSs@Van) that could effectively target D-alanine on the cell walls of bacteria and actively anchored on the bacterial surface [57]. Ma et al. formulated core-shell structures by embedding gold nanorods (GNRs) within layered double hydroxides (LDHSs) [58]. The interaction between the gold component and LDHSs led to an increased electron density on the gold surface, resulting in a PCE of 60 % for GNRs@LDHS, surpassing than other therapeutic materials based on gold nanorods. These GNRs exhibited remarkable efficacy in PTT for wound infections and tissue injuries. The GNRs@LDHS eradicated bacteria on wound surfaces, facilitated the removal of foreign bodies by macrophages, and markedly expedited wound healing in mice. Beyond their wound healing applications, gold nanomaterials also serve as effective delivery vehicles. Peng et al. developed a photothermal system by loading GNRs with bacteriophages and Zn^2+^. Upon NIR irradiation bacteriophages and Zn^2+^ were released. This collaboration effectively improved the sterilization and healing of contaminated wounds [59].

Silver nanomaterials, under the irradiation of near-infrared light, not only generate surface plasmon resonance effects but also release free Ag^+^ ions that bind with sulfur-containing molecules within the bacterial, thereby disrupting bacterial cell division and respiration, resulting in cell death [60]. Silver-based PTAs are a viable option for photothermal antibacterial applications. They display more pronounced and intense plasmon resonance compared to gold nanomaterials, due to increased scattering cross-sections, extinction, and absorption [61]. However, silver nanomaterials are not commonly used in PTT alone due to issues like instability, propensity for aggregation, and potential toxicity. Consequently, scientists adopted hybridization techniques to enhance the precision and effectiveness of silver nanomaterials in PTT antibacterial applications. utilized tannic acid, a naturally occurring antioxidant, as both a reducing and stabilizing agent to coat AgNPs, which were subsequently integrated into a multifunctional hydrogel through crosslinking with an enzymatically catalyzed hyaluronic acid-tyrosine complex [62]. Upon exposure to NIR light, the AgNPs substantially improved the composite hydrogel's antioxidant capacity, bactericidal activity, adhesiveness, and hemostatic capabilities. Agostino et al. employed a seed-mediated growth technique to synthesize citrate-coated triangular silver nanosheets. Upon NIR light exposure, the nanosheets demonstrated significant bactericidal effects against and *E. coli*. and *S. aureus*, leading to the eradication of more than 99.7 % of *E. coli* and around 97 % of *S. aureus* within a 15-min timeframe under NIR illumination [63]. Xie et al. engineered an innovative hybrid coating consisting of GO nanosheets, silver nanoparticles (AgNPs), and collagen. In vivo subcutaneous models demonstrated a synergistic effect between the photodynamic and physical properties of AgNPs within the GO/AgNPs/Col hybrid, enabling swift and efficient bacterial eradication without adverse effects [64]. Wu et al. crafted silver-bismuth nanocomposites on mesoporous silica via an in situ growth method to tackle the challenges of silver clumping and bismuth oxidation [65]. The findings indicated that Ag-Bi@SiO_2_ nanoparticles could enhance the photothermal release of silver ions and mitigate their aggregation. Liu et al. created an efficient antimicrobial hybrid for integrated chemo-photothermal therapy using polydopamine (PDA)-coated GNRs. The PDA layer provided a high loading capacity for silver ions, and subsequent functionalization with glycine-cysteine-serine (GCS) (Ag^+^-GCS-PDA@GNRs) allowed the antimicrobial hybrid to selectively target and concentrate at focal infection sites, showcasing an outstanding synergistic therapeutic impact of chemo-photothermal therapy on abscesses, resulting in complete bacterial elimination, faster wound recovery, and reduced harm to healthy tissues (Fig. 4A-Ⅱ) [66].

Platinum (Pt) nanomaterials serve as viable alternatives to conventional photothermal agents (PTAs) for antibacterial PTT. Deng et al. developed dual-valent Pt (dvPt) nanoparticles, featuring a Pt core and Pt^2+^ shell, for multimodal photothermal antibacterial treatment [67]. Post-NIR irradiation, these engineered dvPt nanoparticles demonstrated exceptional efficacy against methicillin-resistant S.aureus and *E. coli*. Concurrently, the dvPt treatment did not induce systemic toxicity, and the nanoparticles were fully bioresorbable by the renal and hepatic systems, suggesting their potential for clinical use. Furthermore, Pt nanomaterials possess intrinsic catalytic properties that can be synergistically combined with photothermal therapy to amplify the overall antibacterial effect. Zhang et al. prepared a type of Au-Pt nanodots (AuPtNDs) with excellent in vivo biosafety. The Au-Pt nanodots exhibited remarkable PCE and photothermal stability. Upon 808 nm NIR, it could generate hydroxyl radicals, which had a synergistic antibacterial effect, showing good inhibitory effects on both Escherichia coli and S.aureus [68]. Deng et al. have successfully created bivalent platinum nanoparticles (dvPtNPs) for applications in photothermal bactericidal research [69]. These dvPtNPs are composed of a zero-valent platinum core (Pt0) and a divalent platinum shell (Pt2), conferring them with the capability to engage multiple bactericidal mechanisms under near-infrared (NIR) light exposure [70]. The Pt-anchored V_2_CMXene nanoplatforms exhibit versatile properties, such as photothermal and multi-enzyme–like activities, effectively addressing drugresistant bacterial infections. Both in vitro and in vivo studies validate the superior biocompatibility of Pt @V_2_C, along with its remarkable hyperthermia and ROS generation capabilities. Experiments involving subcutaneous abscess and bacterial keratitis demonstrate the outstanding therapeutic potential of Pt@V_2_C, broadening its biological applications beyond traditional chemical catalysis and photo-to-thermal conversion to encompassmodern catalysis and thermal therapy applications (Fig. 4A-Ⅲ).

Palladium (Pd) nanomaterials emerge as promising photothermal agents (PTAs) in photothermal therapy (PTT) for antibacterial purposes, owing to their favorable photothermal stability. Illustratively, upon NIR irradiation, Au-based nanorod PTAs transformed into nanospheres due to gold's low melting point, whereas Pd-based PTAs retained their structure due to palladium's high melting point [71]. Unlike Au-based PTAs, Pd PTAs continued to exhibit robust NIR absorption and a high PCE of 52 %, even when their size was less than 5 nm. This attribute lends Pd-based PTAs greater potential for clinical application, as nanoparticles smaller than 5 nm can be efficiently eliminated by the kidneys. However, in comparison to non-ultra-small scale Au and Ag nanomaterials, Pd nanomaterials typically exhibit a broad and less intense LSPR band in the ultraviolet range and a lower molar extinction coefficient in the NIR spectrum, leading to a comparatively reduced PCE in the NIR region [72]. Consequently, there is a growing emphasis on the modification of Pd-based nanoparticles to enhance their effectiveness in photothermal antibacterial applications. Furthermore, Pd is resistant to deformation at high temperatures induced by NIR irradiation. Both Pd nanosheets(PdNSs) and Pd nanoparticles(PdNPs) have been applied in antibacterial treatments and PTT. Numerous studies have confirmed the bactericidal effectiveness of PdNPs against a range of pathogens, including Enterococcus faecalis, S.aureus, Candida albicans, Escherichia coli, Bacillus cereus, and various Candida species [73,74]. Chen et al. demonstrated that Pd-NPs synthesized via a sustainable and economical microbial reduction method exhibit an extensive absorption spectrum in the NIR region [75]. The application of sonication effectively detaches the majority of Pd-NPs from the microbial host, enhancing their dispersion and consequently their NIR absorption capacity. Additionally, these PdNPs are characterized by superior photostability and biocompatibility. Notably, the microbial-derived Pd-NPs exhibit significant photothermal bactericidal efficacy against both *S. aureus* and Gram-negative *E. coli* even at a minimal concentration of 20 mg/L, underscoring their potential as innovative PTT agents for theranostic purposes (Fig. 4A-Ⅳ).

Ruthenium nanoparticles also have excellent photothermal conversion capabilities, facilitating the transformation of light energy into thermal energy for photothermal therapy to eradicate bacteria. Guo et al. have developed CuRuOX@HA NP antibacterial agents with PTT/CDT through a one-pot process, which enhanced the antibacterial efficacy [76]. These nanoparticles could efficiently eliminate MRSA and *E. coli* through the synergistic action of PTT/CDT in vitro. Moreover, the in vivo therapeutic outcomes on MRSA-infected wounds were positive, with minimal toxicity observed. Crucially, CuRuOX@HA NPs have been shown to prevent the development of bacterial resistance while effectively suppressing the growth of drug-resistant bacteria. This suggests that CuRuOX@HA NPs could serve as a safe and potent antimicrobial strategy against multi-drug-resistant strains, holding promise for clinical use in combating bacterial infections (Fig. 4A-Ⅴ).

##### 2.1.3.2 Transition metal oxide nanomaterials

Nanomaterials derived from metal oxides, including MnO_2_, Fe_3_O_4_, and MoO_3–x_, exhibit strong NIR light absorption due to oxygen vacancies within their crystal structures. These metal oxide nanomaterials offer a more economical and robust alternative to costly noble metal-based PTAs. For instance, Wang et al. developed a NIR-responsive platform containing MnO_2_ (BMH) for the treatment of wounds infected with multidrug-resistant bacteria. In vivo antibacterial efficacy assessments revealed that the BMH hydrogel demonstrated a notable bactericidal effect, achieving a 92.6 % reduction on day 3 and a complete elimination of MRSA by day 14. These findings underscore the BMH hydrogel's potential to effectively counteract multidrug-resistant bacterial infections [77]. Zhu et al. prepared MnO_2_@PDA-BGs/Gel nanocomposite frozen gel, which possesses significant peroxidase-like activity in acidic environments and responds to NIR photothermal stimuli, thereby exhibiting potent antibacterial properties [78]. In vitro cellular assays indicated that MnO_2_@PDA-BGs/Gel enhances the recruitment and proliferation of L929 fibroblasts. Additionally, it mitigates the overproduction of ROS within cells, preserving their viability. In vivo animal studies validated that MnO_2_@PDA-BGs/Gel facilitates wound healing, prevents scarring, and is effective in bacterial eradication, inflammation reduction, vascularization enhancement, and the promotion of collagen III deposition. This gel exhibited strong bactericidal effects against both *S. aureus*, a Gram-positive bacterium, and *E. coli*, a Gram-negative bacterium. The material also showcased exceptional photothermal performance, capable of elevating temperatures to 49.7 °C within a short span of 5 min under a NIR light intensity of 1 W/cm^2^. Upon NIR exposure at 808 nm, KHBP-M achieved a nearly complete (99.99 %) eradication of *S. aureus*, a highly desirable trait for wound treatment, with *E. coli* showing a comparable response. Moreover, the hydrogel excelled in neutralizing ROS and in providing protection against oxidative stress for L929 cells. In studies using animal models with infected wounds, the KHBP-M hydrogel, when subjected to NIR light, displayed the most rapid wound closure, with 82.98 % healing observed by the 15th day [79]. In related work, Zhang and colleagues developed a composite scaffold by embedding Fe_3_O_4_ nanoparticles, approximately 18.4 nm in diameter, into a gelatin matrix. As the concentration of these nanoparticles in the matrix was increased from 1 % to 15 %, the system's temperature rise under 805 nm NIR light at 1.6 W/cm^2^ for 180 s correspondingly increased from 9.3 °C to 26.1 °C [80]. Lv et al. prepared magnetic plasma Fe_3_O_4_@Au core@shell nanocomposite material with 69.9 % photothermal conversion efficiency [81]. The nanocomposites were capable of efficiently eliminating both Gram-negative *E. coli* and Gram-positive *S. aureus* bacteria when exposed to 980 nm laser irradiation, while also exhibiting sustained photostability through multiple rounds of photothermal bacterial inactivation. In a separate study, Zhou et al. discovered that MoO_3–x_ nanobelts, coated with bovine serum albumin, possessed superior photothermal heating capabilities under 1064 nm laser irradiation [82]. Additionally, Wang et al. developed an injectable, adhesive, dual-crosslinked BMH hydrogel that was sensitive to both redox and light triggers, acting as a versatile platform for simultaneous melanoma therapy and healing of wounds infected with multidrug-resistant bacteria by fostering a favorable skin microenvironment (Fig. 4B). [83].

##### 2.1.3.3 Carbon-based nanomaterials

Photothermal agents derived from carbon materials possess several benefits, including high efficiency in converting light to heat, compatibility with biological systems, ease of modification, and affordability. Carbon-derived nanomaterials, including graphene-based nanomaterials GBNs) and CNTs, have attracted considerable attention in PTT because of their unique properties, such as extensive surface areas, superior electronic and thermal conductivities, and impressive optical attributes.

Graphene, a single-layer carbon atom-based two-dimensional nanomaterial, is renowned for its superior mechanical, optical, thermal and electrical characteristics. As a photothermal agent, graphene exhibits remarkable absorption in the NIR spectrum, effectively transforming light into heat [84]. Its antimicrobial capabilities stem from mechanisms such as heat generation, oxidative stress induction, and physical disruption, which are advantageous for preventing infections and aiding in wound recovery. Mei and colleagues notably improved the photothermal performance of nanocomposites by non-covalently attaching PEGylated phthalocyanine to graphene oxide surfaces [85]. Upon 10 min of irradiation, the temperature near the wound tissue in a rat model with bacterial infection rose to 58 °C. The healing rate of the irradiated group was notably higher than that of the control group by the sixth day. Tan et al. prepared RGO/Ag nanocomposites. Under NIR irradiation (0.30 W/cm^2^ 210 min), RGO/Ag nanocomposites significantly improved their synergistic antibacterial ability with the help of photothermal effects. After treatment with RGO/Ag nanocomposites of 15 μg/mL and 30 μg/mL, *E. coli* and Kp were almost completely eliminated. In addition, RGO/Ag nanocomposites showed low cytotoxicity even at lower concentrations, but were sufficient to effectively kill bacteria(Fig. 4C) [86].

Materials like single-walled (SWNT) and multi-walled carbon nanotubes (MWNT) are frequently utilized in antibacterial PTT due to their effective photothermal properties. Hashida et al. created polypeptide-CNT composites with notable photothermal capabilities, which can raise temperatures by over 20 °C within 5 min under near-infrared (NIR) irradiation [87]. Studies have indicated that bacteria exposed to CNTs experience morphological changes, compromised cell membrane integrity, and leakage of intracellular contents, resulting in the loss of bacterial function [88]. By strategic functionalization, CNTs can be engineered to effectively capture bacteria, thus minimizing chemical usage. Yang et al. functionalized CNTs with polyethylene glycol and acrylamide to capture and kill bacteria on wound surfaces, demonstrating high bactericidal efficiency against common pathogens like S.aureus and *E. coli* under NIR irradiation [89]. This approach can adjust wound surface properties based on pH changes, reducing infection risks and promoting healing. This nanoantimicrobial strategy offers innovative non-chemical treatment options and highlights the unique photothermal bactericidal advantages of CNTs. Capitalizing on the antibacterial and photothermal properties of CNTs, various CNT-based photothermal composites have been developed for wound healing. He et al. engineered a conductive, self-healing, and adhesive nanocomposite hydrogel for photothermal therapy of infected wounds, demonstrating notable antibacterial effects against *S. aureus* and *E. coli* under NIR light exposure [90].

##### 2.1.3.4 Metal-organic framework-based materials

Metal-organic frameworks (MOFs), which are porous and crystalline, are created by linking metal ions or clusters with organic ligands. These frameworks are recognized for their substantial surface area and their ability to absorb light and convert it into heat, particularly in the NIR spectrum. This capability allows them to reach high temperatures, which can be lethal to bacteria and can also promote blood vessel growth and cell multiplication [89]. While not all MOFs contain photosensitizers or can be used for photothermal, photocatalytic, or photodynamic applications, some can be modified, for instance, through carbonization or sulfation, to become effective photothermal antibacterial agents [91]. A particular type of MOF, the zeolitic imidazolate frameworks, are known for their capacity to release Zn^2+^ ions. When illuminated, these structures are capable of producing elevated temperatures and demonstrating bactericidal, antioxidant, and anti-inflammatory characteristics [92]. have developed a microneedle patch based on a MOF-organic framework (MN-MOF-GO-Ag) for transdermal drug delivery and combined therapy to enhance diabetic wound healing. The patch, made from a MOF-based antibacterial hydrogel, has been shown to significantly improve the healing process in both in vivo and in vitro studies [93]. Prussian blue (PB), a type of MOF-based material, is known for its biocompatibility and photothermal activity in the NIR range. Chen et al. have synthesized Pb-Yb nanocubes, a MOFs-structured material, where the addition of the rare earth element Yb^3+^ enhances the absorption of NIR light and improves the photothermal conversion efficiency. In vitro tests have demonstrated that PB-Yb exhibits a strong antibacterial effect under 808 nm light irradiation, with an efficacy rate of up to 96 % [94]. Li et al. have developed an exogenous antimicrobial agent made of zinc-doped Prussian blue (ZnPB). Treatment with ZnPB can upregulate genes related to tissue remodeling, promote collagen deposition, and enhance wound repair. The high photothermal conversion efficiency of ZnPB allows it to generate sufficient heat at low irradiation power to deactivate bacteria. Additionally, ZnPB can release zinc ions, which inhibit bacterial growth and biofilm formation, thereby boosting the body's immune response and angiogenesis [19]. Qi et al. have successfully created a photothermally triggered nitric oxide (NO) releasing nanoplatform, PB-NO@PDA-PHMB, for effective bacterial killing. This platform has shown enhanced antibacterial activity against both Gram-positive and Gram-negative bacteria in vitro. Moreover, it could rapidly eliminate *S. aureus* and facilitate wound healing (Fig. 4D).

##### 2.1.3.5 MXenes

MXenes are a class of two-dimensional transition metal materials that consist of carbides, nitrides, or carbonitrides, characterized by a chemical formula of M_n+1_X_n_T_x_, where 'M' denotes an early transition metal, 'X' is either carbon or nitrogen, and 'T' represents a surface group. These newly emerged 2D inorganic materials, such as Ti_3_C_2_, Ti_2_C, Ti_2_N, Mo_2_C, and Nb_2_C, are recognized for their long-range in-plane order and anisotropic crystal structure, making them ideal candidates for photothermal agents. They are known for their high PCE, favorable light absorption, and ultrathin electronic properties. The synthesis of MXenes typically involves an etching process in a fluoride-rich acidic medium, like hydrogen fluoride [95]. Li et al., have synthesized a Ti_2_N-based nanosystem with an impressive PCE of 41.6 %, capable of increasing the temperature of a liquid solution by 15 °C within 3 min under 808 nm NIR irradiation [96]. Feng et al. developed an ultrathin Mo_2_C MXene with strong NIR absorption across both the first (650–1000 nm, NIR-I) and second (1000–1350 nm, NIR-II) bio-transparent windows. When incorporated into a polyvinyl alcohol matrix, this hybrid system showed a PCE of 24.5 % for NIR-I and 43.3 % for NIR-II [97]. Zheng et al. created a novel thermo-sensitive hydrogel (Cip-Ti_3_C_2_ TSG) that combines the antibiotic ciprofloxacin (Cip) with Ti_3_C_2_ MXene nanocomposites. This hydrogel acts as a promising platform for targeted drug delivery and improved antibacterial action, featuring high PCE and the capacity to boost the release of Cip under NIR light exposure [98]. Hao et al. proposed a strategy involving the immune activation of neutrophils using a DNase I-loaded vanadium carbide MXene (DNase-I@V_2_C) [99]. This material effectively scavenges ROS in highly oxidative environments, preserving the activity of DNase I. DNase-I@V_2_C can penetrate deep into biofilms, degrading extracellular DNA and neutrophil extracellular traps (NETs), thereby disrupting biofilm structure. Furthermore, by modulating immune switch points, DNase-I@V_2_C can redirect neutrophil function towards phagocytosis, breaking down biofilms. This approach has shown promising therapeutic efficacy, highlighting the potential of modulating immune switch points to influence neutrophil activities and treat drug-resistant bacterial infections (Fig. 4E).

### 2.2 Photosensitizers for antibacterial PDT

#### 2.2.1 Organic near-infrared photodynamic nanomaterials

##### 2.2.1.1 Porphyrin

**Fig. 5:**
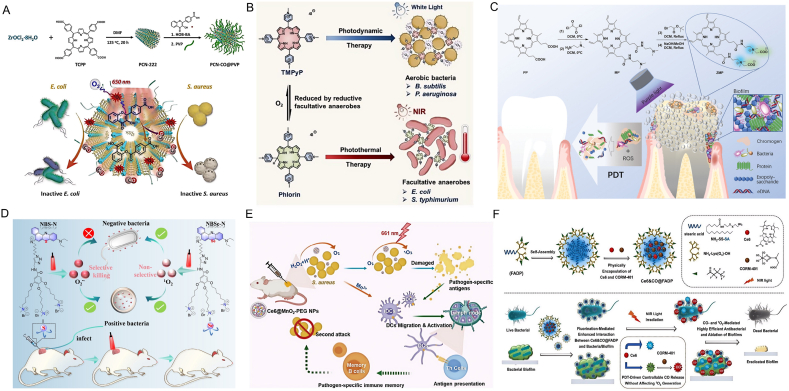
Antibacterial application of organic photosensitizers. (A) A diagrammatic representation of the red light-triggered liberation of singlet oxygen (^1^O_2_) and carbon monoxide (CO) through a photooxygenation process in PCN-CO@PVP, showcasing superior bactericidal activity. Reproduced with permission from Ref. [105]. Copyright 2023,The Royal Society of Chemistry. (B) Schematic presentation of the porphyrin TMPyP that responds to bacterial stimuli for adaptable photodynamic and photothermal treatment. Reproduced with permission from Ref. [106]. Copyright 2022,Wiley-VCH GmbH. (C) Synthetic route for ZMP and a diagram showing the application of PDDT for dental whitening and the removal of dental biofilms. Reproduced with permission from Ref. [108]. Copyright 2021,Wiley-VCH GmbH. (D) Schematic illustration of ROS generated by NBS-N and NBSe-N, along with the targeted elimination of bacteria and the wound recovery in infected mice. Reproduced with permission from Ref. [113]. Copyright 2022, Wiley-VCH GmbH. (E) Schematic presentation of Ce6@MnO_2_-PEG-based PDT for managing bacterial infections. Reproduced with permission from Ref. [116]. Copyright 2020, Wiley-VCH GmbH. (F) A diagrammatic depiction of the synthesis of Ce6&CO@FADP and the antibacterial action. Reproduced with permission from Ref. [119]. Copyright 2020,American Chemical Society.

Derivatives of porphyrin, characterized by their tetrapyrrole structures connected via methane bridges, represent a second-generation of photosensitizers [102]. Their optimal light absorption in the visible spectrum, rapid transition to the triplet state, substantial quantum efficiency, and structural flexibility render them as prime contenders for antiphotodynamic therapy (aPDT). aPDT involves the generation of ROS through the interaction of light, photosensitizers, and adjacent molecules to eradicate harmful microorganisms, with a well-understood mechanism [103,104]. As pioneers in this field and with the distinction of being the first photosensitizers sanctioned by the U.S. FDA, porphyrins have been broadly applied across various domains, exhibiting promise in both oncological and antimicrobial applications. Zhang et al. synthesized an innovative composite material based on a porphyrin-derived metal-organic framework, designated PCN-CO@PVP [105]. This material demonstrated a swift reaction to irradiation at 650 nm, enabling the emission of ROS and CO through the photooxygenation of HOB-BA, catalyzed by ^1^O_2_ produced by PCN. At low concentrations, PCN-CO@PVP displayed remarkable antibacterial properties, particularly effective against Gram-positive bacteria like *S. aureus* and MRSA. Mechanistic studies revealed that PCN-CO@PVP had a more pronounced affinity for *S. aureus* than for *E. coli*, which enhanced its selective bactericidal impact (Fig. 5A). Hu et al. engineered engineered a porphyrin-based compound, TMPyP, designed to be responsive to bacteria and suitable for versatile photodynamic and photothermal therapies tailored to specific environmental conditions. When Staphylococcus aureus, Bacillus subtilis, and Pseudomonas aeruginosa were exposed to TMPyP under white light, the findings indicated an exceedingly high inhibition rate of 99.9 % for these bacterial species (Fig. 5B). [106]. Yu and colleagues crafted an acid-activated amphiphilic block copolymer, POEGMA-b-[PDPA-co-PTPPC6MA], which self-assembled into nanoparticles known as PDPA-TPP. These nanoparticles exhibited rapid disintegration in the acidic conditions of bacterial microenvironments, with a pH of 5.5 [107]. This pH-responsive nanoplatform improved binding to bacterial cell membranes and mitigated the aggregation-caused quenching (ACQ) effect associated with porphyrin-based photosensitizers. This intervention led to a significant enhancement in ^1^O_2_ generation, increasing it by a factor of five (Fig. 5C) [108]. The distinctive electron donor-acceptor framework along with the aqueous solubility of ZMP substantially amplified its capacity to generate ROS, exhibiting a potent bactericidal impact on S.aureus and Streptococcus mutans. Ji and colleagues explored the bactericidal efficacy of cationic porphyrin derivatives against Pseudomonas aeruginosa. Their research revealed that these cationic porphyrins, under the influence of photodynamic antibacterial chemotherapy (PACT), inflicted more substantial damage on Pseudomonas aeruginosa. This treatment effectively disrupted the bacterium's microstructure, leading to its inactivation and demise [109].

##### 2.2.1.2 Phenothiazine

Phenothiazines, categorized as cationic dyes, have emerged as noteworthy antimicrobial photosensitizers, attributed to their pronounced light absorption in the 600–800 nm range [110]. Methylene blue (MB), a monocationic phenothiazine with a tricyclic π-system featuring auxochromic substituents, stands out as an established therapeutic agent for chronic periodontitis and oral mucositis. It has also shown effectiveness in photo-activated plasma disinfection against antibiotic-resistant strains and viruses. Adjustments to the phenothiazine framework may yield enhanced photosensitizing properties, with the majority of research thus far concentrating on peripheral substitutions of the tricyclic structure, the incorporation of additional aromatic or alicyclic hydrocarbons into the fused rings, and the replacement of sulfur atoms within the central molecular framework [111]. Gao and team pioneered the synthesis of phenothiazine-based analogs through a pyridine fusion approach, achieving a significant reduction in S.aureus by a factor of 10^6 at a concentration of 0.5 μM (0.21 ng/mL) and in Escherichia coli by a factor of 10^5 at 6 μM (2.52 ng/mL) [111]. Balhaddad and colleagues engineered a multifunctional photosensitizer nanoplatform termed MagTBO, which integrates TBO with superparamagnetic iron oxide nanoparticles (SPIONs) using the microemulsion technique [112]. With the aid of an external magnetic field, MagTBO could deeply infiltrate the biofilm, markedly reducing the bacterial count in Streptococcus mutans biofilms by 10^6, thereby illustrating the potential of magnetic navigation to augment the bactericidal impact of photosensitizers. Yoon's research group developed two phenothiazinium-based photosensitizers, NBS-N and NBSe-N, sharing an identical core structure but with distinct elemental compositions (sulfur vs. selenium) (Fig. 5D) [113]. NBS-N, generating superoxide anion (O_2_^•-^), selectively eliminated *S. aureus* over *E. coli*, whereas NBSe-N, producing ^1^O_2_, showed robust bactericidal activity against both. This differential effect may stem from the conversion of O_2_^•−^by NBS-N into the highly •OH predominantly within bacterial cells, whereas the highly toxic ^1^O_2_ generated by NBSe-N can effectively damage bacteria across the outer membrane barrier.

##### 2.2.1.3 Chlorophyll

Chlorophyll-based photosensitizers represent a second-generation class, structurally distinct from porphyrin-based counterparts. This group encompasses photosensitizers such as chlorophyll-a, pheophorbide a, p-bro-mo-phenylhydrazone-methyl pyropheophorbide-a, temoporfin, and chlorin e6 (Ce6). Notably, Ce6 stands out for its efficacy in PDT applications; however, its advancement is impeded by challenges related to low aqueous solubility and susceptibility to clearance [114]. Yan et al. prepared UHSN@CS-Ce6 material that can destroy mature S.aureus biofilms, resulting in an 81 % reduction in biomass [115]. Wang et al. engineered nanoparticles named Ce6@MnO_2_-PEG, capable of regulating the abscess microenvironment, boosting the efficacy of PDT for skin abscesses, and stimulating the immune response [116]. These nanoparticles consist of MnO_2_, which is doped with the photosensitizer Ce6 and coated with PEG. The MnO_2_ component is designed to break down excess H_2_O_2_ into O_2_ within the abscess environment, thereby enhancing PDT effectiveness (Fig. 3E). To counteract the oxygen-deficient conditions at infection sites, Qu's team developed CeCyan-Cu_5.4_O, a construct that integrates oxygen-generating cyanobacteria-supported photosensitizer Ce6 with ultrasmall Cu_5.4_O nanoparticles exhibiting catalase properties, aiming to combat anaerobic infections [117]. Ma et al. created Ce6&CO@FADP, a system that utilizes a fluorinated amphiphilic dendritic peptide (FADP) to encapsulate the Ce6 photosensitizer and the CO prodrug (CORM-401) [118]. The fluorination of FADP promotes the adherence of Ce6&CO@FADP to bacterial cells and facilitates O_2_ supply for PDT. Upon bacterial internalization, Ce6&CO@FADP swiftly liberates CO within the cells by utilizing H_2_O_2_ generated during PDT. This combined strategy of photodynamic therapy (PDT) and carbon monoxide (CO) gas treatment exerts a significant synergistic impact on the eradication of biofilms linked to subdermal bacterial infections and catheter-associated biofilm models. Ma et al. have successfully introduced an innovative PDT-activated controlled CO delivery system, Ce6&CO@FADP, for potent antibacterial action and biofilm disruption (Fig. 3F) [119]. The FADP's fluorination enhances Ce6&CO@FADP's bacterial binding and O_2_ provision for PDT. Once inside bacteria, Ce6&CO@FADP quickly releases CO by metabolizing H_2_O_2_ generated during PDT. The fusion of PDT with CO gas therapy offers a pronounced synergistic effect in the eradication of biofilms in subcutaneous infections and catheter-associated biofilm models.

##### 2.2.1.4 BODIPY and its derivatives

BODIPY dyes are widely acclaimed for their structural diversity and excellent spectral properties. By functional modification of BODIPY derivatives, they can be endowed with various photophysical properties, which opens up broad prospects for their application in the field of antibacterial. Shi et al. synthesized six BODIPY derivatives (BDP1-BDP6), and the results showed that BDP3 showed the highest ROS production capacity, which could effectively eliminate Staphylococcus aureus with a minimum inhibitory concentration of 10 nM and prevent the development of bacterial biofilms. With its high antibacterial performance, excellent biofilm inhibition effect and good biocompatibility, BDP3 shows broad prospects as a potential PS candidate to solve bacterial infections [120]. Zhao et al. prepared a BodiPy-based glycosylation photosensitizer (pGEMA-I), which showed good water solubility and excellent ROS production capacity. Gram-negative bacteria (*P. aeruginosa*), normal cells (NIH3T3), and their mixed cultures were treated with pGEMA. The results showed that *P. aeruginosa* exhibited bright green fluorescence, which was hardly detected in the NIH3T3 cells, indicating that it was able to selectively bind to *P. aeruginosa* rather than to normal cells, thereby effectively eliminating the pathogen by producing ROS. After 60 min of light exposure, almost all bacteria lost their ability to reproduce. At the same time, the experimental results indicate that pGEMA-I can significantly inhibit the growth of Pseudomonas aeruginosa. In addition, when pGEMA-I concentration is 800 μg/mL, the biofilm inhibition ability can reach 90 % [121]. Aza-BODIPY derivatives have the advantages of high molar extinction coefficient, excellent light stability, adjustable photophysical properties, simple synthesis process, high phototoxicity to dark toxicity ratio and redshift absorption and emission spectra [122]. Yang et al. prepared a novel halogen-free photosensitizer Aza-BODIPY-BODIPY binary NDB NPs with orthogonal molecular configuration. Under 730 nm laser irradiation, NDB NPs has excellent ROS generation capability. In addition, in vitro antibacterial experiments showed that NDB NPs could effectively inactivate methicillin-resistant *S. aureus* [123].

#### 2.2.2 Inorganic nano photosensitizers

##### 2.2.2.1 Metal nanoparticles

Metal nanoparticles made of metals, with sizes between 10 and 100 nm, feature a large surface area relative to their volume. This attribute allows for effective engagement with bacterial biofilms, leading to their destruction. Commonly used metal nanoparticles include gold and silver nanoparticles. Gold nanoparticles are easily shape-manipulable and can be designed into nanoclusters, nanospheres, nanorods, and nanoflowers, among others, according to specific needs. Additionally, they exhibit localized surface plasmon resonance under light irradiation [124].

##### 2.2.2.2 Metal oxide nanoparticles

**Fig. 6:**
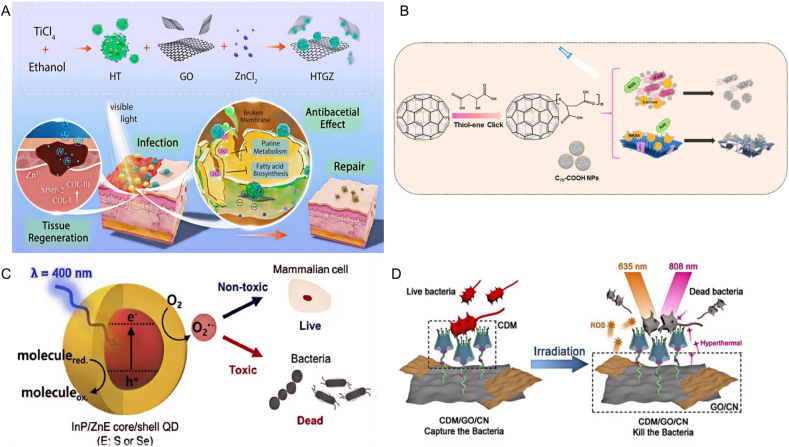
Antibacterial application of inorganic nano photosensitizers.(A) A diagram illustrating the HTGZ nano-system, which possesses an enhanced photodynamic antibacterial effect and the capacity to expedite the healing of infected wounds. Reproduced with permission from Ref. [134] (B) Schematic representation of the preparation of C70-COOH NPs and their antibacterial and anti-biofilm photodynamic activity. Reproduced with permission from Ref. [136]. Copyright 2022,Elsevier B.V [138]. (C) A diagrammatic depiction of photodynamic therapy facilitated by the quantum dots. Reproduced with permission from Ref. [150]. Copyright 2022,he Royal Society of Chemistry. (D) A diagrammatic representation of the configuration and bactericidal mechanism of CDM/GO/CN. Reproduced with permission from Ref. [151]. Copyright 2022,American Chemical Society.

Nanoparticles of metal oxides, such as zinc oxide and titanium oxide, are biocompatible semiconductors known for their distinctive optical characteristics. Under light irradiation, they generate highly reactive free radicals, exhibiting broad-spectrum bactericidal effects [125]. Commonly used metal nanoparticles include gold and silver nanoparticles. Gold nanoparticles are easily shape-manipulable and can be designed into nanoclusters, nanospheres, nanorods, and nanoflowers, among others, according to specific needs. TiO_2_ NPs, as PSs in PDT can generate ROS for bactericidal treatment [126]. Wang et al. investigated the synergistic antibacterial impact of a titanium dioxide/nano-hydroxyapatite (TiO_2_-HAP) composite material on dental biofilms. Their study revealed that the application of the TiO_2_-HAP composite under LED illumination nearly eradicated Streptococcus mutans biofilms. This treatment not only led to the near-complete removal of the biofilm but also caused significant damage to the bacterial cell membranes, resulting in a roughened cellular appearance. These findings underscore the material's potent bactericidal properties [127]. Su et al. developed a nanocomposite pharmaceutical formulation, designated as TiO_2_/curcumin/hydroxypropyl cyclodextrin (TiO_2_/Cur/HPCD), and integrated it with konjac glucomannan to fabricate composite films known as KTCHD films [128]. These films demonstrated favorable biocompatibility and potent antibacterial properties, attributed to their PDT effects. The TiO_2_/Cur/HPCD component was found to adsorb onto bacterial surfaces, thereby enhancing the therapeutic efficacy. In vitro antibacterial tests on TiO_2_/Cur/HPCD revealed that the material lacked antibacterial activity in the absence of light. However, its bactericidal capacity escalated with increasing concentrations under illuminated conditions. Specifically, at a concentration of 500 μg/mL, the survival rate of MRSA was significantly reduced to 25.54 %. In vivo studies validated the efficacy of TiO_2_/Cur/HPCD and KTCHD films in treating bacterial infections and accelerating wound healing. Notably, the KTCHD-10 membrane combined with light irradiation achieved the highest wound closure rate, peaking at 84.6 % by the twelfth day of the study. Magesan et al. synthesized Fe_2_O_3_-TiO_2_ (FT) with PVP-PEG-assisted nanocomposites, wihch had greater photodynamic activity potential. In addition, the nanocomposites showed significant antibacterial activity against Escherichia coli [129]. Wang et al. crafted a TiO_2_/TiO_2−x_ metasurface with robust NIR responsive antibacterial properties for Ti alloy implants, utilizing a novel alkaline-acid bidirectional hydrothermal method [130]. This metasurface, under low-power NIR irradiation, produced ^1^O_2_ and •OH, leading to significant antibacterial effects. The in vitro antimicrobial efficacy against *E. coli* and *S. aureus* reached 96.88 % and 97.56 %, respectively, while the in vivo antimicrobial rate against *S. aureus* was 91.8 %. ZnO, a semiconductor metal oxide with distinctive optical characteristics, generates ROS through electron transfer, thus exhibiting antimicrobial properties. Wu et al. developed Ag/Ag@AgCl/ZnO composite nanostructures incorporated into a hydrogel, which improved photocatalytic bactericidal capabilities [131]. The ESR relative intensity from the Ag/Ag@AgCl/ZnO nanocomposite hydrogel was markedly higher than that of the pure ZnO hydrogel, suggesting that the incorporation of Ag/Ag@AgCl enhanced the generation of hydroxyl radicals and ^1^O_2_. Upon visible light exposure, this hydrogel eradicated 95.95 % of *E. coli* and 98.49 % of *S. aureus* in vitro, and the release of Zn^2+^ ions accelerated wound healing in vivo. In addition to altering ZnO nanoparticles, the synthesis method also plays a role in determining their photocatalytic antibacterial performance [132]. Ma et al. developed a multifunctional hydrogel dressing, incorporating gelatin (Gel), sodium alginate (SA), transglutaminase (mTG), and calcium ions, modified with black phosphorus nanosheets (BPNS), forming a three-dimensional cross-linked network. In vitro antimicrobial tests showed that the antibacterial rates for S.aureus and *P. aeruginosa* were approximately 90.6 ± 3.1 % and 96.4 ± 3.3 %, respectively, in groups treated with 100BP@ZnO-Gel/SA and exposed to NIR [133]. Lu et al. created a highly hydrophilic and uniformly dispersed TiO2-based nano-system known as HTGZ, which showed improved photothermal properties. HTGZ exhibited outstanding antibacterial activity, resulting in a greater than 98 % decrease in both S. mutans and *E. coli* populations, and displayed good cytocompatibility in vivo (Fig. 6A) [134].

##### 2.2.2.3 Fullerenes

Fullerenes, including C60, C70, C84, and others, form molecular cages that generate ROS upon exposure to light, reaching a triplet state. These fullerenes and their derivatives, particularly the cationically functionalized forms, exhibit a wide-ranging photodynamic antimicrobial effect against both bacteria and fungi by generating superoxide anions and free radicals [135]. They offer several advantages over traditional photosensitizers (PSs), such as increased resistance to photobleaching, a higher overall ROS quantum yield, and the capacity to self-assemble into nanoparticles. Nonetheless, it is essential to modify their surfaces to enhance their solubility in water. A recently developed photosensitizer (PS) based on C70, synthesized in a single-step thiol reaction, can produce a substantial quantity of ROS through both type I and type II mechanisms, causing bacterial cell membrane disruption. The type I reaction, independent of oxygen, produces cytotoxic ROS such as •OH, superoxide anions (O_2_^•-^), and H_2_O_2_, whereas the type II reaction, oxygen-dependent, primarily generates ^1^O_2_). These luminescent C70-COOH nanoparticles are capable of deactivating various bacterial strains, including MRSA [136]. Incorporating additional functional groups (such as COOH, OH, and NH_2_) into fullerenes enhances their aqueous solubility and boosts their ROS generation potential. For example, the water-soluble C60(OH)30 fullerene alcohol, with a high superoxide anion quantum yield of 0.89, has been shown to produce significant ROS and is effective against multidrug-resistant bacteria [137]. Zhang et al. created an efficient photodynamic hydrogel through the self-assembly of a small peptide and a fullerene, suitable for targeted and sustained antibacterial therapy [138].The hydrogel benefits from a combination of non-covalent interactions, including hydrogen bonding, π-π stacking, hydrophobic interactions, and electrostatic repulsion, which lead to a synergistic enhancement of the hydrogel's properties. The peptide nanofibers encapsulate the fullerene as nanoparticles, thereby increasing the fullerene's singlet oxygen generation activity. Tan et al. have devised an ingenious and straightforward strategy for the one-step synthesis of C70-based photosensitizers through thiol-ene reactions. The resulting C70-COOH NPs exhibit exceptional anti-photobleaching properties and photostability in aqueous media. The outcomes of cytotoxicity and hemolysis tests robustly demonstrate their excellent biocompatibility. Upon exposure to visible light, C70-COOH NPs steadily generate reactive oxygen species (ROS) through Type I and Type II mechanisms, producing sufficient free radicals even under oxygen-deprived conditions. Consequently, they inflict irreversible damage on both Gram-positive and Gram-negative bacteria, particularly against Staphylococcus aureus and MRSA. Notably, the photodeactivation of C70-COOH NPs also effectively prevents the formation of MRSA biofilms. This offers a novel perspective for C70 derivatives as potential broad-spectrum antimicrobial photosensitizers (Fig. 6B) [136].

##### 2.2.2.4 Quantum dots

Quantum dots (QDs) possess the ability to absorb photons and subsequently emit light at wavelengths longer than the excitation source, making them potential candidates for PS delivery. However, early generations of QDs exhibited cytotoxic effects. Graphene quantum dots, which have the capacity to emit ROS, serve as effective PSs [139]. Their biocompatibility and solubility in water can be improved through surface modifications [140]. For instance, Kuo et al. enhanced the functionality of nitrogen-doped graphene quantum dots by introducing amino groups, creating a photosensitizer that effectively eradicated MRSA in vitro following photodynamic therapy with a 670-nm laser, outperforming non-modified graphene quantum dots [141]. Carbon dots (CDs) represent a promising class of nano-photosensitizers [142]. Their positive charge allows them to penetrate bacterial membranes. When excited by visible or near-infrared light, they can generate ROS through a triplet energy transfer mechanism [143]. CDs are characterized by their high photostability, biocompatibility, and the potential to be combined with other antimicrobial agents to amplify their bactericidal effects. Carbon dots (CDots), spherical carbon-based fluorescent nanoparticles typically under 10 nm in size, have become a novel platform for antimicrobial agents activated by visible or natural light [144]. These CDots offer a safer alternative to conventional photosensitizers (PSs), boasting non-toxicity, quantum confinement effects, and a high capacity for drug loading. Wei et al., for instance, created carbon quantum dots (CQDs) from citric acid and 1,5-diaminonaphthalene in an ethanol solution and assessed their efficacy as PSs for in vitro photodynamic inactivation of a range of bacterial strains, both Gram-positive and Gram-negative [145]. Under visible light, these CQDs produced ^1^O_2_ through a specific pathway, leading to the inactivation of *E. coli* and *S. aureus*. Although the ROS they generate can potentially compromise the photostability of the CDots, striking a balance is essential for their modification. To address this, Das et al. covalently attached BSA to CDots. After the amine groups of BSA modified the surface carboxyl groups of CDots, it significantly enhanced ROS generation [146]. GQDs represent a category of carbon-based nanomaterials characterized by exceptional physicochemical attributes such as photostability, solubility, a small size of around 10 nm, the capacity for multiphoton excitation, and the ease of surface modification [147]. These traits render GQDs suitable for various applications, including acting as photosensitizers, biosensors, and components of drug delivery systems. The synthesis approach for GQDs significantly influences their physicochemical characteristics; for instance, electrochemical techniques produce highly uniform GQDs, whereas oxidative techniques result in particles with a high degree of oxidation. When activated by light, GQDs as photosensitizers can produce substantial quantities of ROS [147,148]. Their large surface area and the possibility of surface functionalization allow for the efficient loading of additional photosensitizers onto the GQDs, enhancing their photoactivity [149]. The structure of GQDs can be readily tailored to boost their photodynamic performance; for example, nitrogen doping of GQDs improves light absorption and extends the photoluminescence lifetime, thereby optimizing ROS generation [147]. Lee et al. utilized InP/ZnSe QDs for both therapeutic and prophylactic management of MRSA-infected skin wounds in a mouse model, demonstrating the efficacy of InP/ZnSe QDs in vivo [150]. They found that a 15-min PDT session with these QDs was adequate to achieve full sterilization of the wounds. No adverse effects from PDT were detected, affirming the safety of the O_2_^•-^ mediated approach to combat MRSA using InP QDs. The findings indicate that InP QDs have the potential to act as antibacterial agents in the treatment and prevention of bacterial infections. This innovative strategy may help address the challenge posed by the increasing prevalence of antibiotic-resistant bacteria (Fig. 6C).

##### 2.2.2.5 Other photosensitizers

Graphite carbon nitride (g-C_3_N_4_), a member of the two-dimensional non-metal semiconductor nanomaterials, is highly valued for its beneficial properties like a substantial surface area, biocompatibility, stability, lack of toxicity, a moderate band gap, chemical resistance, and a characteristic layered structure. It has become a global focus in areas such as photocatalysis, energy transformation, electrochemistry, and bioimaging. Among various photosensitizers, g-C_3_N_4_ is seen as a candidate with promise due to its favorable energy band alignment, attractive optical properties, and its unique characteristics as an n-type semiconductor. fabricated a hybrid material consisting of β-cyclodextrin-derivative-modified graphene oxide and graphitic carbon nitride, known as CDM/GO/CN, which combines mannose-modified β-cyclodextrin (CDM), GO, and g-C_3_N_4_ [151]. The incorporation of CDM enhances the water solubility of the GO/CN mixture and minimizes its tendency to aggregate. Moreover, the mannose component has an affinity for capturing specific strains of *E. coli*, such as CICC 20091. The composite leverages the synergistic effects of PDT and PTT from g-C_3_N_4_ and GO, demonstrating exceptional antibacterial activity with an efficacy rate exceeding 99.25 % against *E. coli* following a 10-min exposure to dual-wavelength light at 635 and 808 nm. This CDM/GO/CN material is anticipated to offer innovative solutions in the biomedical sector for antibacterial applications, with promising potential in managing bacterial infections, preventing infections associated with medical device implants, and serving in wound care dressings (Fig. 6D). Kang et al. developed porous g-C_3_N_4_ nanoflakes, which were observed to produce ROS upon light exposure, leading to the elimination of bacteria [152]. Li et al. created a g-C_3_N_4_ composite film that demonstrated bactericidal properties when stimulated by light [153]. However, the rapid recombination of electrons and holes, coupled with the dense structure of bacterial biofilms, can diminish the antibacterial efficacy of PDT. Recently, black phosphorus (BP), recognized as one of the most stable phosphorus allotropes, has been employed as a photosensitizer when exfoliated into ultra-thin 2D nanosheets [154]. Their adjustable bandgaps, elevated carrier mobility, and robust absorption of light across the visible to NIR spectrum make BP nanoplates (BPANP) proficient producers of ^1^O_2_, positioning them as prime contenders for PDT. BP's potential for eradicating bacterial cells is notable, largely due to its capacity to generate singlet oxygen species. Shaw et al. have documented that BP, when thin, triggers ROS production, instigating cellular oxidation and subsequent lysis [155]. Consistent with this, microbial cells treated with BP nanosheets (BPNS) for 2 h exhibit significant membrane damage, with the majority unable to survive. Liu et al. have validated that BP can efficiently generate ROS, which oxidizes the phospholipid bilayer of bacteria, culminating in bacterial demise [156]. Upon light exposure, BP excites an electron and transfers energy to oxygen, leading to ^1^O_2_ formation. In bacterial presence, this ^1^O_2_ targets the bacterial phospholipid layer. Zhang et al. introduced an innovative metal-free 2D heterostructure, BP-CN, combining BP with graphite carbon nitride (CN), achieving complete bacterial inactivation within 60 min [157]. They also developed BP/Cu nanocomposites to augment antibacterial properties through interfacial charge transfer, leveraging BP's unique electronic traits to boost ROS production [158]. Cong et al. prepared a chitosan-black phosphorus (CS-BP) hydrogel, which, upon exposure to simulated sunlight for only 10 min, demonstrated remarkable antimicrobial efficacy against *E. coli* and S.aureus, at rates of 98.90 % and 99.51 %, respectively. This efficacy is primarily due to the swift generation of dense ROS by BP post-irradiation [159]. Both in vitro and in vivo findings indicate that BP within this hybrid hydrogel can stimulate early fibrinogen formation, facilitating the crusting phase of tissue repair. Tan et al. detailed an antimicrobial membrane made from black phosphorus nanosheets (BPSs) and poly(4-pyridine-methylstyrene) endoperoxides (PPMS-EPO), which controls ROS storage and release in a reverse manner [160]. In vitro antibacterial studies revealed that the PMS-EPO/BPS film possessed a swift disinfection capability, with antibacterial rates of 99.3 % against Escherichia coli and 99.2 % against S.aureus after a 10-min irradiation period. Hematoxylin and eosin (H&E) staining of mouse tissues confirmed the membrane's excellent biocompatibility in vivo.

## 3 Photosensitizers with synergistic antibacterial effect of PTT and PDT

### 3.1 Antibacterial effect of the synergy between PTT and PDT

The combination of PDT and PTT in a single photosensitizer offers a synergistic effect that can overcome the limitations of each individual therapy. For instance, the heat generated by PTT can enhance the uptake of photosensitizers and increase ROS generation, leading to a more rapid and effective elimination of bacteria. Additionally, the thermal effect can disrupt bacterial biofilms, which are often resistant to traditional antibiotics and PDT alone.

#### 3.1.1 Organic nanomaterials with PPT and PDT properties

**Fig. 7:**
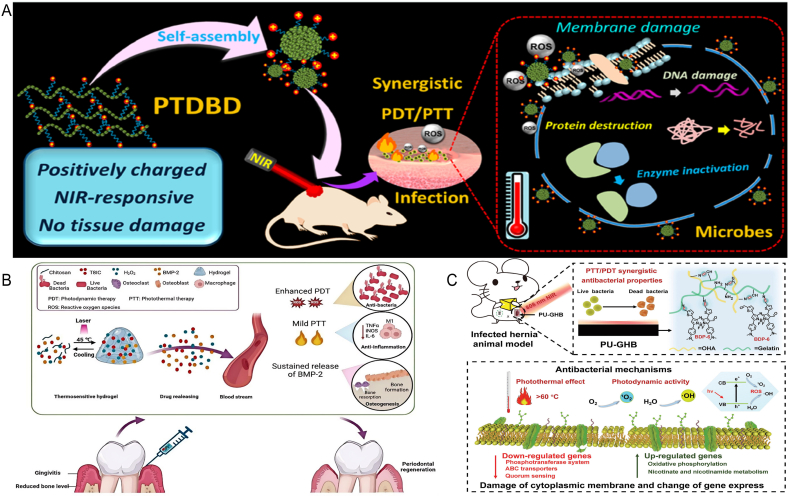
Application of organic nanomaterials in combination with PTT and PDT for antibacterial activity.(A) A diagrammatic depiction of the synthesis of PTDBD and its mechanisms of photothermal and photodynamic therapy for antibacterial and anti-infective applications. Reproduced with permission from Ref. [163]. Copyright 2020,American Chemical Society. (B) Schematic presentation of a hydrogel that was both injectable and temperature-responsive, harnessing nano-enhanced NIR-II photothermal and chemical therapeutic properties for combating periodontal bacteria and promoting bone tissue regeneration. Reproduced with permission from Ref. [167]. (C) A diagrammatic depiction of the characteristics and operational mechanisms of PU-GHB. Reproduced with permission from Ref. [168].

Photosensitizers with both PPT and PDT therapeutic effects are a special type of material that can not only generate heat under light irradiation, but also produce ROS. The combined photodynamic and photothermal antibacterial approach, utilizing a single light source, simplifies the treatment process and shows significant promise for combating bacterial infections. Zhou et al. have engineered a NIR activated antibacterial strategy employing the cationic conjugated polymer PTDBD, which delivers both PDT and PTT effects [163]. The inclusion of quaternary ammonium groups in the polymer's side chains promotes engagement with microbial cells, boosting the generation of ROS and thermal energy upon illumination, both of which are detrimental to the microorganisms. With a minimal light intensity of 1.0 W cm^−2^ at 808 nm and a brief exposure of 8 min, this method effectively eradicated three types of microbes: Ampr *E. coli* (Gram-negative), *S. aureus* (Gram-positive), and C. albicans (fungal), at a concentration not exceeding 60.0 μg/mL. Moreover, this therapeutic approach was successfully tested on S. aureus-infected wounds in mice, with no significant harm to healthy tissues observed (Fig. 7A). Conjugated polymers (CPs) possess remarkable potential in the biomedical field due to their distinctive π-conjugated backbones and superior photophysical characteristics [164,165]. A key benefit of CPs is their capacity to produce ROS and thermal energy upon light irradiation, a feature that can be tailored by modifying their backbone structures. Moreover, CPs exhibit biocompatibility, photostability, and are cost-effective to synthesize. Consequently, they have become promising materials for PDT and PTT. Cui et al. created an antibacterial hydrogel based on a water-insoluble conjugated polymer (PDPP) that possesses combined PTT and PDT effects [166]. This polymer intelligently modulates its bactericidal activity when exposed to white light followed NIR light, demonstrating enhanced bactericidal efficacy compared to either PDT or PTT alone. Zhang et al. employed nanoparticles composed of a dual-mode antibacterial conjugated polymer (DM-CPNs) to effectively eliminate ampicillin-resistant *E. coli* through PTT and PDT [164]. Near infrared light irradiated DMCPNs group for 5min, the temperature was as high as 62.4 °C, showing excellent photothermal ability. Further studies showed that DMCPN could sensitize surrounding oxygen under light, thus generating reactive oxygen species. The DM-CPNs achieved a 93 % inhibition rate against ampicillin-resistant *E. coli* following simultaneous exposure to near-infrared light (at 550 mW/cm^2^ for 5 min) and white light (at 65 mW/cm^2^ for 5 min). Wang et al. created an injectable, temperature-sensitive hydrogel featuring 3D networks that serve as a drug delivery system for the regulated discharge of osteogenic factor (BMP-2) and NIR-II phototherapy components (T8IC NPs and H_2_O_2_) (Fig. 7B). [167]. In the in vitro antibacterial test, solely green fluorescence was detected in the control group, as well as in the groups treated with Hydrogel, Hydrogel + H_2_O_2_, and Hydrogel + BMP-2. The biofilms exhibited near-complete red staining in the Hydrogel + T8IC + Laser + BMP-2 + H_2_O_2_ (45 °C) group, demonstrating a significant bactericidal effect. Wang and colleagues engineered an injectable, adhesive, dual-crosslinked BMH hydrogel that is responsive to redox and light, serving as a versatile platform for the concurrent treatment of melanoma and the healing of wounds infected with multidrug-resistant bacteria (Fig. 7C) [168]. Song et al. prepared Aza-BODIPY(ABDP) nanoparticles, which have good photothermal effect and excellent ROS generation ability. Under 808 nm laser irradiation, ABDP nanoparticles showed that the killing efficiency of PTT and PDT on Escherichia coli and MRSA reached more than 99.9 %. In the wound healing experiment, on day 14, neat arrangements of epidermal structures were observed only in the ABDPNPs + NIR irradiated group. Hair follicle regeneration can be observed with H&E staining, while Masson staining shows more skin components such as collagen. In addition, the experimental results showed that the formation of excessive inflammatory environment was avoided by PDT/PTT dual-mode collaborative sterilization [169]. Liu et al. prepared a urease-driven bowl shaped dopamine mesoporous nanorobot (MPDA@ICG@Ur@Man) for the synergistic antimicrobial therapy of PTT and PDT. The results show that it has good photothermal conversion ability and good ^1^O_2_ production ability. During the PTT process, the local temperature increase can enhance the activity of urease, which helps to enhance the autonomous movement of bacteria, and thus increase the contact chance of antibacterial substances with bacteria. Biofilm irradiated with 808 nm NIR laser for 8 min showed the strongest elimination effect in the MPDA@ICG@Ur@Man + NIR group [170].

#### 3.1.2 Inorganic nanomaterials for both PTT and PDT therapy

##### 3.1.2.1 Metal sulfide nanomaterials

**Fig. 8:**
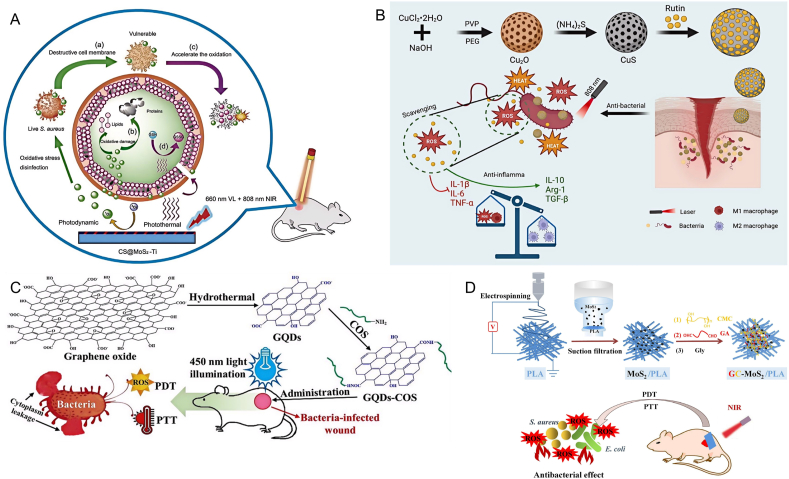
Application of inorganic nanomaterials in antimicrobial therapy of PTT and PDT. (A) An in vivo sterilization approach that targets bacterial cell membranes and neutralizes bacteria using the photodynamic and photothermal properties of the CS@MoS_2_ hybrid coating on titanium implants, facilitated by the application of two light sources. Reproduced with permission from Ref. [182]. Copyright 2018,WILEY-VCH Verlag GmbH & Co. KGaA, Weinheim. (B) Fabrication of CuS@Rutin and its mechanism of bacterial inhibition, as well as its regulation of macrophage polarization to address inflammation caused by bacterial infections. Reproduced with permission from Ref. [174].(C) A diagrammatic depiction of the synthesis of GQDs-COS and their utilization in antibacterial purposes and the management of wounds infected by bacteria. Reproduced with permission from Ref. [192]. Copyright 2020,American Chemical Society.(D) A schematic representation of the fabrication process for GC-MoS_2_/PLA composite nanofiber mats designed for synergistic photothermal and photodynamic antibacterial therapy. Reproduced with permission from Ref. [193].Copyright 2024 Elsevier B.V.

Metal sulfide nanomaterials are widely used because of their relatively simple preparation method, low price, outstanding photothermal effect and good biocompatibility. CuS is a typical metal sulfide used for antibacterial purposes. CuS nanoparticles have emerged as a prospective contender for photothermal therapy due to their inherent near-infrared absorption, ability to generate heat efficiently, cost-effectiveness, biodegradability, and the versatility offered by their gold nanoshells. These nanoparticles are valuable not only for their role as photosensitizers in PTT, but also for their capacity to generate ROS during PDT under NIR light exposure. In addition, due to the stimulating effect of copper ion on cell proliferation and angiogenesis, it has been demonstrated the ability to facilitate wound recovery. Therefore, the system based on CuS nanoparticles may provide antibacterial ability while promoting wound healing. Dai et al. synthesized poly (5-(2-ethyl acrylate)-4-methylthiazole-*g*-butyl)/CuS nanoclusters, leveraging the combined effects of PDT and PTT to effectively target and neutralize levofloxacin-resistant bacteria [171]. Thiazole derivatives were applied to the nanocomposites as membrane-targeted cationic ligands for bacteria. When exposed to NIR laser irradiation (980 nm, 1.5 W/cm^2^, 5 min), the aforementioned conjugated nanoclusters substantially suppressed the growth of Amylobacter, *E. coli*, levofloxacin-resistant S.aureus, and *P. aeruginosa*. The antibacterial effect was credited to the heat and ROS generated by the NIR light, signifying a combined effect from both PTT and PDT. Mutalik et al. established that the shape of CuS determines its photodynamic and photothermal antibacterial effects under NIR light against *E. coli* [172]. In a comparable investigation, Zhou and colleagues introduced an injectable, self-healing hydrogel incorporating a CuS complex, which was able to completely eradicate *E. coli* and *S. aureus* via the synergistic effects of PTT and PDT under NIR laser irradiation [145]. Along the same lines, Qiao et al. demonstrated the bactericidal capabilities of CuS nanodots against MRSA and extended-spectrum β-lactamase *E. coli*, employing a PTT and PDT strategy under NIR laser exposure [173]. Liu et al. Synthesized CuS@Rutin nanoparticles. Upon treatment with near-infrared laser irradiation, the combination of CuS@Rutin with PTT and PDT exhibited an enhanced, cooperative antibacterial action. Additionally, CuS@Rutin facilitated the beneficial shift in macrophage polarization from the pro-inflammatory M1 type to the anti-inflammatory M2 type [174]. This modulation of macrophage polarization may aid in curbing inflammatory responses, thereby potentially enhancing the resolution of bacterial infections (Fig. 8B). Li et al. synthesized CuS particles with flower-like nanostructures (CuS@Al), which showed good PTT and PDT properties. *E. coli* and S.aureus were inactivated under 0.5 mg/ml CuS@Al [175]. Liu et al. prepared a multifunctional nano-antimicrobial agent BCPP for targeted PDT/PTT combination therapy. BCPP is composed of photosensitizer PpIX, photothermal agent CuS, targeted fragment PBA and colloidal stabilizer BSA. The experimental results show that BCPP has a better ability to produce 1O2 than porphyrins alone. After irradiation with the NIR laser (808 nm, 1.0 W cm ^2^,10 min), the temperature increase is concentration-dependent, showing excellent photothermal capacity. In vitro antibacterial experiments showed that the antibacterial rate of BCP in the PDT/PTT combined treatment group was 92 % for *S. aureus*, *E. coli*. The antibacterial rate was 90 %. The effect was better than that of single treatment group [176].Xu et al. developed MoS_2_/PLA composite nanofiber [177]. Upon heating to 55 °C, these mats exhibited heightened antibacterial potency, with efficacy rates climbing to 94.7 % against S.aureus and 90.1 % against Escherichia coli. This suggests that the dual application of heat and ROS can significantly bolster the antibacterial properties. Moreover, under NIR radiation, the inhibitory effects on S.aureus and *E. coli* became even more pronounced, with rates escalating to 98 % and 97.1 %, respectively. Xia et al. have successfully designed a intelligent antibacterial nanoplatform that integrated MoS_2_ with Cu_3_Mo_2_O_9_ [178]. When the sample concentration was 100 μg/mL, the temperature of the microenvironment near bacteria was increased rapidly from 28.6 °C to more than 50 °C within 6 min NIR.This temperature elevation significantly enhanced cell membrane permeability, facilitating the internalization of ROS into bacterial cells. Post-treatment, the antibacterial rate against Escherichia coli could surpass 99 %. Wang et al. prepared Fe_3_O_4_@MoS_2_@SDS nanocomposites [179], which exhibited a rapid increase in fluorescence absorbance within the initial 6 min of NIR exposure, plateauing after 10 min. These nanocomposites demonstrated superior ∙OH radical generation capabilities. In wound healing assessments, a marked reduction in wound area was observed in the Fe_3_O_4_@MoS_2_@SDS (1:1) + NIR group, with approximately a 3-log10 decrease in bacterial load post-treatment compared to the PBS control group. Shen et al. engineered bacterial cellulose/MoS_2_–chitosan nanocomposites for PTT and PDT antibacterial functions, showcasing remarkable efficacy against *E. coli* (99.998 %) and *S. aureus* (99.988 %) following visible-light exposure [180]. Yougbare et al. synthesized MoS_2_@AuNR nanocomposites. After 10 min of 808 nm NIR irradiation, the solution temperature of MoS_2_@AuNR nanocomposites increased from 25 °C to 66.7 °C, showing superior photothermal effect. MoS_2_@AuNR nanocomposites are also excellent at producing ROS under visible light irradiation. After near infrared laser and visible light irradiation, MoS_2_@AuNRs can achieve complete bactericidal effect, indicating that the antibacterial effect of PTT and PDT is better than that of single therapy [181].Wang et al. enhanced the antibacterial properties of biomedical titanium implants by leveraging the photodynamic and photothermal characteristics of a CS@MoS_2_ hybrid coating under simultaneous short-term dual light (660 nm VL + 808 nm NIR) irradiation [182]. CS molecules were noncovalently affixed to MoS_2_ sheets, preventing their re-aggregation. These CS-decorated MoS_2_ nanodispersions, produced via ionic liquid-assisted grinding, were then applied to Ti plate surfaces using electrophoresis, with the resulting sample named CS@MoS_2_-Ti. The dual irradiation with visible light VL and NIR light activated the coating's potent bactericidal effect against both *E. coli*, and *S. aureus* in a short period (Fig. 8A). Xu et al. prepared GC-MoS_2_/PLA antibacterial nanofiber dressings. This dressing successfully integrated functional nanoparticles, carried drugs in a stable manner, and it has been confirmed that there is a significant synergy between PTT and PDT. MoS_2_ itself possesses catalytic activity, enabling the composite material to spontaneously generate reactive oxygen species. Under near-infrared light irradiation, the favorable photothermal effect of MoS_2_ exhibits higher catalytic capacity. The GC-MoS_2_/PLA dressing causes the bacterial cell membrane to become permeable, resulting in protein efflux and an increase in intracellular reactive oxygen species (ROS) levels. In the in vitro antibacterial experiments, compared with single hyperthermia (with almost no antibacterial activity) or ROS treatment, the GC-MoS_2_/PLA dressing demonstrates significant antibacterial effects against Staphylococcus aureus (*S. aureus*) and Escherichia coli (*E. coli*) (Fig. 8D).

##### 3.1.2.2 Carbon-based materials

Carbon-based materials are highly regarded for their suitability in biomedical uses due to their minimal toxicity and eco-friendly characteristics. Their customizable features, including size, shape, and layer count, endow them with superior physiochemical properties, making them exceptional for antibacterial purposes. Materials like carbon dots and graphene have been particularly investigated for their potential in combined photothermal and photodynamic therapy approaches, leveraging their capacity to convert NIR light into heat. The broad success of graphene in medical applications is evident, with an abundance of research focusing on its central role. The distinctive light-absorbing and heat-releasing capabilities of graphene and its variants, such as GO and RGO, have spurred their adoption in PTT [[183], [184], [185]]. Moreover, upon NIR light exposure, graphene can generate ROS. It's also posited that graphene's razor-sharp edges can penetrate bacterial cell walls, resulting in the demise of the bacteria [186].The wide-ranging antibacterial effects of GO and RGO have been harnessed by researchers. One notable benefit of graphene lies in its capacity to be designed into a versatile nanocomposite capable of performing both photodynamic and photothermal antibacterial functions. Sun and colleagues described the up-conversion nanoparticles crafted from NaYF_4_: Yb: Tm nanorods featuring a multi-layered core-shell structure (UCNPs@TiO_2_) [187]. Then, GO was integrated into the up-conversion nanoparticles to form a UTG nanocomposite, which was then put into a PVDF matrix, yielding a UTG–PVDF nanocomposite. When exposed to a NIR source at 980 nm for 5 min, it simultaneously induce ROS generation and temperature increase, exerting a combined antibacterial impact against both Gram-positive and Gram-negative bacteria. Moreover, the film hastened the wound healing process, boosting the potential for healing wounds infected by pathogens. Romero and colleagues demonstrated the extensive and specific bactericidal action of GO and nGO against bacteria through the combined impacts of photothermal and photodynamic therapies [184]. GO and nGO were able to produce ROS under exposure to an LED red light, efficiently inactivating the targeted bacteria through combined effects of PDT and PTT. The photosensitizer ICG, commonly employed in PDT, was deposited onto photothermal GO nanosheets to boost the antibacterial activity against methicillin-resistant S.aureus under NIR irradiation [185]. ICG–GO achieved a temperature elevation of 43.1 °C and a significant ROS production, rendering it an effective agent for curbing the proliferation of MRSA. This was integrated into magnetic nanocomposites that include GO and carbon nanotubes (CNTs) to create a biocompatible hydrogel [188]. This nanocomposite was effective against C. albicans, *E. coli*, and *S. aureus* by a combined PDT and PTT effect. Liu et al. combined BP exfoliated in liquid phase with tellurium-doped CDs prepared by hydrothermal method to construct a two-dimensional antibacterial nano-platform, thus amalgamating the beneficial biomedical attributes of CDs with the photothermal properties of BP [189]. The photothermal conversion of BP was triggered by 808 nm NIR irradiation, and ROS was produced by CDs. BP@CDs showed remarkable synergistic antibacterial activity against *E. coli* and *S. aureus* through PTT and PDT (up to 98.4 and 92.7 %, respectively), and the wound healing rate was superior. The BP@CD nano-platform demonstrated good blood compatibility, cytocompatibility, and biocompatibility. Chu and colleagues developed a blend of copper ions with CDs and quaternary ammonium salts to attain a synergistic PPT and PDT for bacterial therapy on *E. coli* and *S. aureus* under NIR irradiation, reaching a peak temperature of 57.3 °C and efficiently producing ROS [190]. Fang and colleagues developed nitrogen-doped carbon quantum dots loading with curcumin, which displayed effective photodynamic and photothermal synergistic antibacterial effects against the *E. coli* and *S. aureus*, enhancing curcumin's photokilling efficacy against bacteria. Moreover, these composite nanomaterials exhibited minimal toxicity and favorable blood compatibility [191]. Mei et al. prepared GQDs-COS and investigated the phototherapy effects in a rat model of bacterially infected wounds. GQDs-COS could facilitate rapid wound healing from bacterial infection. Observation of rat dermal wounds stained with H&E and immunohistochemistry indicated that GQDs-COS treatment could resolve inflammation caused by bacterial infection. The nanocomposite also demonstrated good blood compatibility and low cytotoxicity for infected wound healing (Fig. 8C) [192].

## 4 Effect of phototherapy on wound microenvironment

Phototherapy has a significant impact on the wound microenvironment, influencing various aspects of wound healing including immune response, cellular activity, and the overall healing process. As the core element of PDT, ROS can not only destroy the structural integrity of bacteria, cause the leakage of bacterial contents, but also irreversibly damage the double helix structure of bacterial DNA [194].ROS induced microbial cell structure damage and cell death due to lipid peroxidation can further activate neutrophils, phagocytes, dendritic cells (DCs) and T cells. These activated cells then secrete cytokines, chemokines and other bioactive mediators that contribute to wound healing [195].Studies have shown that after PDT treatment of wounds, the inflammatory response is effectively controlled during the healing process, and the healing speed is significantly accelerated [196,197].This effect is partly due to the activation of the immune system and the optimization of the local microenvironment, which promotes the wound to return to its normal function more quickly. The increase in temperature in PTT promotes blood circulation, affects enzyme activity, and activates cell signaling, ultimately contributing to tissue repair, thereby improving the microenvironment for wound healing. On the other hand, moderate photothermal therapy can enhance the expression of heat shock proteins (HSPs) [198], This not only improves the cells' tolerance to heat stress, but also further activates the immune system and promotes the healing of wounds in vivo. Chen et al. designed CaSiO_3_-ClO_2_@PDA-ICG (CCPI) nanoparticles for the PTT/PDT combination treatment of biofilm wound healing. By studying the maturity of DCs (CD80 + CD86 +) cells, they found that the maturity of DCs cells increased significantly after CCPI + NIR treatment, and the study results showed that CCPI NPs enhanced the migration ability of tissue cells in the wound area by regulating the phenotype of macrophages, thus promoting the wound healing process. In the exploratory wound healing experiment, the reduction of wound area in the CCPI + NIR treatment group was significantly greater than that in the other control group. Down-regulation of pro-inflammatory cytokine IL-6 and up-regulation of platelet endothelial cell adhesion molecule-1 (CD31) suggest that CCPI therapy has anti-inflammatory and wound healing effects. Comparing the wound healing effect of animals with different immune states, studies have shown that the antibacterial effect and wound healing effect achieved by PDT/PTT combination therapy are largely dependent on the activated immune system, especially the role of M2 macrophages [199]. Kong et al. prepared MGC NPs to achieve efficient wound healing through immune activation and macrophage autophagy promotion. The results show that L-H-MGC + NIR treatment can effectively activate the immune microenvironment of wound tissue during wound treatment, effectively regulate local inflammatory response, promote the transformation of macrophages from M1 phenotype to M2 phenotype, and thus accelerate the wound healing process. The upper layer of the hydrogel contains a high concentration of MGCNPs, which can release a large number of ROS to remove microorganisms at the wound site under the irradiation of 808 nm laser, while the lower layer of hydrogel contains a lower concentration of MGCNPs and releases a small amount of ROS under the irradiation of 808 nm laser. Low levels of ROS can enhance the autophagy function of M2-type macrophages by promoting the increased expression of IL-1β and TLR-1. These M2-type macrophages with increased expression and improved autophagy ability can accelerate the movement of epithelial cells at the wound site by promoting the secretion of TGF-β1, thus promoting wound healing. This indicates that PDT/PTT combined therapy is of great significance for wound healing [200]. Yang et al. developed a variety of stimulation-responsive nanozymo-based Cryogels (C/N/MPA Cryogels), which have been used as a controlled NO release system for adaptive treatment of infectious wound healing. Under the irradiation of 808 nm NIR, the surface temperature of cryogels increased significantly after adding MSPA(PDA, L-Arginine functionalized MOS_2_ composition), which indicates that MSPA has excellent photothermal conversion efficiency. Compared with pure DPBF without MSPA, the absorption peak of cryogels with MSPA was significantly reduced at the wavelength of 410 nm. In addition, with the increase of MSPA content, the absorption peak also decreases, and this reduction is proportional to the concentration of MSPA, suggesting that the cryogels have a high efficiency in producing ^1^O_2_. Under NIR irradiation, MSPA produces a large amount of ROS through energy transfer, which further catalyzes the conversion of L-arginine to L-citrulline and NO. In vitro antibacterial experiments, after 3 min of NIR irradiation, the bactericidal efficiency of *E. coli* and MRSA reached 98.4 % and 98.5 % respectively. When the irradiation time was extended to 5 min, the bactericidal efficiency of the two bacteria reached almost perfect 100 %. In vivo antibacterial experiment, a subcutaneous abscess model of MRSA-infected mice was established. After 3 days of treatment, the number of inflammatory cells in cryogels group was significantly reduced. MSPA shows PDT and PTT effects in response to NIR, and can interact with NO to enhance the killing effect of bacteria. In the physiological environment without NIR, MSPA as a nano-enzyme can promote the clearance of excess ROS, thereby improving the wound microenvironment. In the process of evaluating the healing of infected wounds, C/N/MPAs promoted the rapid accumulation of collagen at the wound site and contributed to the repair and regeneration of damaged blood vessels, thereby accelerating wound healing [201].

## 5 Conclusion and prospective

Phototherapy, a non-invasive treatment approach, is recognized for its minimal impact on healthy tissues and its reduced toxic side effects compared to traditional antimicrobial chemotherapy. This modality's direct bacterial targeting results in limited side effects and avoids systemic adverse reactions such as disruptions to the gut microbiota or damage to the liver and kidneys. Photodynamic therapy (PDT) harnesses reactive oxygen species (ROS) generated by the interaction of light with photosensitizers, effectively neutralizing a broad spectrum of pathogenic bacteria, including antibiotic-resistant strains like Staphylococcus aureus and Porphyromonas gingivalis. The efficacy of PDT is due to the potent oxidative action of ROS, which swiftly disrupts bacterial membranes by damaging phospholipids, proteins, and DNA, thereby halting bacterial proliferation and inducing cell death. Photothermal therapy (PTT), on the other hand, utilizes agents that convert light energy into heat, inducing localized hyperthermia and bacterial inactivation. These features render phototherapy a promising frontier with substantial development potential in antimicrobial treatments. This review summarizes various nanomaterials involved in photothermal or photodynamic therapy for antibacterial purposes, including organic and inorganic materials. Finally, nanomaterials with synergistic photothermal and photodynamic therapeutic effects were summarized. In general, an ideal photosensitizer for clinical application should meet the following requirements: (1) Small "hidden toxicity" and side effects in the body; (2) It exhibits strong absorbance within the phototherapy window (650–900 nm), resulting in good PTT effects or prominent ROS generation efficiency for PDT; (3) Excellent photostability and biocompatibility, allowing for rapid clearance from the body. By synthesizing the current literature, future research directions are identified, with a focus on enhancing photothermal conversion efficiency, deepening tissue penetration, and improving the biocompatibility and biodegradability of nanomaterials. Addressing the key challenge of improving nanomaterial photothermal conversion efficiency involves material selection, structural design, and optimization to boost carrier concentration and optical properties. Strategies such as hollow nanocage design to broaden illumination range and shape control to modulate light absorption are being investigated. Surface modifications to enhance light absorption and thermal stability through molecular coating or grafting are highlighted. The collective application of these strategies is anticipated to markedly enhance the photothermal conversion efficiency or ROS generation rate of nanomaterials. Ongoing research is aimed at augmenting treatment efficacy and biosafety by integrating phototherapy with complementary therapeutic modalities, including antibiotics, sonodynamics, gas therapy, and nano-enzymes. These innovations offer fresh perspectives for phototherapy in antibacterial applications, holding the promise of delivering significant social and economic benefits to public health.

## CRediT authorship contribution statement

**Ling Mei:** Writing – review & editing, Writing – original draft, Conceptualization. **Yifan Zhang:** Writing – original draft. **Kaixi Wang:** Writing – review & editing, Investigation. **Sijing Chen:** Writing – review & editing. **Tao Song:** Writing – review & editing, Project administration.

## Declaration of competing interest

The authors declare that they have no known competing financial interests or personal relationships that could have appeared to influence the work reported in this paper.

## Data Availability

No data was used for the research described in the article.
